# Low-Value Care at the Actionable Level of Individual Health Systems

**DOI:** 10.1001/jamainternmed.2021.5531

**Published:** 2021-09-27

**Authors:** Ishani Ganguli, Nancy E. Morden, Ching-Wen Wendy Yang, Maia Crawford, Carrie H. Colla

**Affiliations:** 1 Division of General Internal Medicine and Primary Care, Brigham and Women’s Hospital, Harvard Medical School, Boston, Massachusetts; 2 The Dartmouth Institute for Health Policy and Clinical Practice, Geisel School of Medicine, Lebanon, New Hampshire

## Abstract

**Importance:**

Low-value health care remains prevalent in the US despite decades of work to measure and reduce such care. Efforts have been only modestly effective in part because the measurement of low-value care has largely been restricted to the national or regional level, limiting actionability.

**Objectives:**

To measure and report low-value care use across and within individual health systems and identify system characteristics associated with higher use using Medicare administrative data.

**Design, Setting, and Participants:**

This retrospective cohort study of health system–attributed Medicare beneficiaries was conducted among 556 health systems in the Agency for Healthcare Research and Quality Compendium of US Health Systems and included system-attributed beneficiaries who were older than 65 years, continuously enrolled in Medicare Parts A and B for at least 12 months in 2016 or 2017, and eligible for specific low-value services. Statistical analysis was conducted from January 26 to July 15, 2021.

**Main Outcomes and Measures:**

Use of 41 individual low-value services and a composite measure of the 28 most common services among system-attributed beneficiaries, standardized to distance from the mean value. Measures were based on the Milliman MedInsight Health Waste Calculator and published claims-based definitions.

**Results:**

Across 556 health systems serving a total of 11 637 763 beneficiaries, the mean (SD) use of each of the 41 low-value services ranged from 0% (0.002%) to 41% (7%) of eligible beneficiaries. The most common low-value services were antibiotics for acute upper respiratory and ear infections (mean [SD] rate, 41% [7%] of eligible beneficiaries), preoperative laboratory testing (mean [SD] rate, 28% [4%] of eligible beneficiaries), prostate-specific antigen testing in men older than 70 years (mean [SD] rate, 27% [8%]), and use of antipsychotic medications in patients with dementia (mean [SD] rate, 23% [8%]). In multivariable analysis, the health system characteristics associated with higher use of low-value care were smaller proportion of primary care physicians (adjusted composite score, 0.14 [95% CI, 0.03-0.25] for systems with less than the median percentage of primary care physicians vs −0.15 [95% CI, –0.26 to –0.04] for those with more than the median percentage of primary care physicians; *P* < .001), no major teaching hospital (adjusted composite, 0.11 [95% CI, 0.0 to 0.22] without a teaching hospital vs −0.21 [95% CI, –0.37 to –0.05] with a teaching hospital; *P* = .003), headquartered in the South or West (adjusted composite, 0.27 [95% CI, 0.13-0.42] for the South and 0.15 [95% CI, –0.05 to 0.36] for the West compared with −0.09 [95% CI, –0.26 to 0.08] for the Northeast and −0.39 [95% CI, –0.54 to –0.23] for the Midwest; *P* < .001), and serving areas with more health care spending (adjusted composite, 0.25 [95% CI, 0.14-0.37] for areas above the median level of spending vs −0.26 [95% CI, –0.38 to –0.14] for areas below the median level of spending; *P* < .001).

**Conclusions and Relevance:**

The findings of this large cohort study suggest that system-level measurement and reporting of specific low-value services is feasible, enables cross-system comparisons, and reveals a broad range of low-value care use.

## Key Points

**Question:**

How does low-value care use vary across health systems?

**Findings:**

This cohort study measured and reported the use of 41 individual low-value services and a composite measure of 28 services for 556 health systems serving a total of 11 637 763 Medicare beneficiaries across the US. Systems varied widely in the provision of low-value care; those with a smaller proportion of primary care physicians, without a major teaching hospital, headquartered in the South and West, and serving areas with higher health care spending delivered more low-value care.

**Meaning:**

This study suggests that claims-based definitions can be used to measure low-value care within systems, providing granular, actionable feedback to promote health care quality and affordability.

## Teaser

This cohort study uses Medicare data to measure and report low-value care use across and within individual health systems and identify system characteristics associated with higher use.

## Introduction

Low-value health care services are still widely delivered in the US despite decades-long efforts to curb them through measurement and reporting.^1^ The continued use of these services creates health and cost burdens for patients and society.^2^ The COVID-19 pandemic interrupted some low-value services (along with high-value care), but paths to sustaining these lower rates remain unclear.^3^ This is partly because low-value care has largely been measured and reported at the national or regional level, limiting accountability and actionability.

Actionable metrics are critical to the deimplementation of low-value care.^4^ Once delivery patterns are established, they are slow to change, especially if the service capability reflects investments in technology, infrastructure, or the workforce and generates substantial revenue.^5,6^ Changing clinical practice requires adopting new approaches and deimplementing existing practices^7,8^ with the help of timely, specific, and personal feedback to change behavior at the individual or organizational level.^9,10^ Although frameworks now exist to promote the deimplementation of low-value care, such methods require granular measurement to be effective.^11,12^

To address this gap in actionable measurement and reporting, we used widely accepted, claims-based definitions and attribution methods to measure use of 41 distinct low-value services and a composite low-value care measure for beneficiaries attributed to 556 individual health systems across the US. We then identified health system characteristics associated with higher use of low-value services.

## Methods

### Data and Population

For this cohort study, to generate health system–level low-value care measures, we used 2015-2017 Medicare fee-for-service administrative data from the Beneficiary Summary file, claims and administrative records (Inpatient, Medicare Provider Analysis and Review, Outpatient, Part B, Home Health Agency, Part D, Durable Medical Equipment, and Skilled Nursing Facility), the Long Term Care Minimum Data Set, and First Databank. We examined the care of beneficiaries older than 65 years of age who were enrolled for at least 12 months or until death in Medicare Parts A and B in either 2016 or 2017. For select measures, we also used a random 40% sample of fee-for-service prescription (Part D) data. For measures using prescription information for eligibility exclusions, we required Part D enrollment for all months (≥12 months or until death) of Parts A and B enrollment. For measures in which prescription receipt was an outcome, we required at least 1 month of Part D enrollment. We excluded members with any hospice claims in 2016-2017. To determine health system and area-level characteristics, we used the Agency for Healthcare Research and Quality 2016 Compendium of US Health Systems (AHRQ Compendium), IQVIA OneKey, and MedInsight developed by Milliman (formerly Leavitt Partners’ Torch Insight).^13,14,15^ This study was approved by the Dartmouth Committee for the Protection of Human Subjects. We received permission from IQVIA to report individual health system performance. To comply with Centers for Medicare & Medicaid Services reporting restrictions, we reported ranges of the number of beneficiaries attributed to each health system and suppressed system-level, low-value measure results with fewer than 11 beneficiaries.^16^

### Health System Identification and Attribution

We identified health systems from the AHRQ Compendium. These systems are relatively large and include a range of workforce compositions, reimbursement models, and organizational structures. The AHRQ Compendium defines systems as including at least 1 hospital and at least 1 group of physicians providing comprehensive care; the hospital(s) and physician group(s) are linked through common ownership or joint management.^13^

We linked Medicare beneficiary claims data to health systems by attributing each beneficiary to a single health system based on the plurality of primary care services received across 2016 and 2017, a method adapted from previous analyses.^17^ We restricted our analyses to systems that were not predominantly pediatric (based on name) and with 250 or more attributed beneficiaries.

### Low-Value Care Measures

We operationalized 41 claims-based, low-value care definitions described in prior research and in the Milliman MedInsight Health Waste Calculator, a stand-alone, proprietary software program that identifies potentially inefficient services based on recommendations from the Choosing Wisely campaign and professional medical societies (Table 1).^18^ The calculator flags services as necessary, likely to be wasteful, and wasteful; to be conservative, we included only the wasteful services. Two physician researchers (I.G. and N.E.M.) reviewed measures to identify 36 Milliman low-value care definitions relevant to our study population. We defined and coded 5 additional measures described in previous studies.^19,20,21,22,23^ For each measure, we identified the beneficiaries eligible (ie, at risk) for the potentially low-value service within each health system and counted eligible beneficiary-months as relevant for the measure until death or December 31, 2017, to capture the time during which a beneficiary had the opportunity to receive a given low-value service. We then calculated service use within each health system, defined as the number of eligible beneficiary-months of those who received a given service at least once divided by all eligible beneficiary-months in 2016-2017.

**Table 1.** Low-Value Service Measure Descriptions

Category and key No.	Label	Description
Laboratory testing		
1^a^	Preoperative laboratory testing	Do not perform baseline laboratory studies in patients without significant systemic disease (ASA I or II) undergoing low-risk surgery
2^a^	PSA testing	Do not perform PSA-based screening for prostate cancer in men older than 70 years
3^a^	25-Hydroxy vitamin D testing	Do not perform population-based screening for 25-hydroxy-vitamin D deficiency
4	Testing for chronic urticaria	Do not routinely do diagnostic testing in patients with chronic urticaria
5^a^	Immunoglobulin G or E testing	Do not perform unproven diagnostic tests, such as immunoglobulin G testing or an indiscriminate battery of immunoglobulin E tests, in the evaluation of allergy
6	Bleeding time testing	Do not use bleeding time testing to guide patient care
Imaging		
7^a^	Imaging for eye disease	Do not routinely order imaging tests for patients without symptoms or signs of significant eye disease
8^a^	Short-interval repeat DEXA scan	Do not routinely repeat DEXA scans more often than once every 2 years
9^a^	Imaging for headache	Do not perform imaging for uncomplicated headache
10^a^	Carotid artery imaging for simple syncope	Do not perform imaging of the carotid arteries for simple syncope without other neurologic symptoms
11^a^	Head imaging for syncope	Do not obtain brain imaging studies (CT scans or MRI) in the evaluation of simple syncope and a normal neurologic examination
12^a^	Emergency department head CT scan for dizziness	Do not perform routine head CT scans for emergency department visits for dizziness
13^a^	Imaging for low back pain	Do not perform imaging for low back pain within the first 6 weeks unless red flags are present
14^a^	Head CT scan for sudden hearing loss	Do not order CT scan of the head or brain for sudden hearing loss
15^a^	Imaging for uncomplicated acute rhinosinusitis	Do not routinely perform radiographic imaging for patients who meet diagnostic criteria for uncomplicated acute rhinosinusitis
16	MRI for rheumatoid arthritis	Do not perform MRI of the peripheral joints to routinely monitor inflammatory arthritis
17	Coronary artery calcium scoring for known CAD	Do not use coronary artery calcium scoring for patients with known CAD (including stents and bypass grafts)
18	DEXA scan in low-risk patients	Do not use DEXA screening for osteoporosis in women younger than 65 years or men younger than 70 years with no risk factors
Cardiopulmonary and neurologic testing		
19^a^	Screening ECGs	Do not order annual ECGs or any other cardiac screening for low-risk patients without symptoms
20^a^	Preoperative ECG, chest radiographs, or PFT	Do not perform ECGs, chest radiographs, or PFT in patients without significant systemic disease (ASA I or II) undergoing low-risk surgery
21^a^	EEG for headaches	Do not perform EEG for headaches
22^a^	Cardiac stress testing	Do not perform stress cardiac imaging or advanced noninvasive imaging in the initial evaluation of patients without cardiac symptoms unless high-risk markers are present
23	PFT prior to cardiac surgery	Do not recommend PFT prior to cardiac surgery in the absence of respiratory symptoms
24	Preoperative echocardiography or cardiac stress testing	Do not perform baseline diagnostic cardiac testing or cardiac stress testing in asymptomatic stable patients with known cardiac disease undergoing low- or moderate-risk noncardiac surgery
Procedures		
25^a^	Cervical cancer screening	Do not order unnecessary cervical cancer screening (Papanicolaou test and human papillomavirus test) in all women who have had adequate prior screening and are not otherwise at high risk for cervical cancer
26^a^	Injection for low back pain	Do not provide outpatient epidural, facet, or trigger point spinal injections for low back pain
27^a^	Repeat short-interval colorectal cancer screening	Do not order unnecessary screening for colorectal cancer in adults older than 50 years
28^a^	Peripheral access placement without nephrology consultation in stage III-V CKD	Do not place peripherally inserted central catheters in patients with stage III-V CKD without consulting nephrology
29	Feeding tubes for patients with dementia	Do not recommend percutaneous feeding tubes for patients with advanced dementia
30^a^	PCI for asymptomatic patients	Avoid PCI for stable, asymptomatic patients with normal or only mildly abnormal adequate stress test results
31^a^	Vertebroplasty for osteoporotic fractures	Do not perform vertebroplasty for osteoporotic vertebral fractures
32^a^	Coronary angiography in low-risk patients	Do not perform coronary angiography in patients without cardiac symptoms unless high-risk markers are present
33	Multiple palliative radiotherapy treatments for bone metastases	Do not recommend more than a single fraction of palliative radiotherapy for an uncomplicated painful bone metastasis
34	Renal artery revascularization	Do not perform revascularization without prior medical management for renal artery stenosis
35	Arthroscopic lavage and debridement for knee osteoarthritis	Do not perform an arthroscopic knee surgery for knee osteoarthritis
Drugs		
36^a^	Antipsychotics for patients with dementia	Do not use antipsychotics as first choice to treat behavioral and psychological symptoms of dementia
37^a^	Opiates for acute disabling low back pain	Do not prescribe opiates for acute disabling low back pain before evaluation and a trial of other alternatives is considered
38^a^	Antibiotics for acute upper respiratory tract and ear infections	Do not prescribe oral antibiotics for patients with upper respiratory tract or ear infection (acute sinusitis, viral respiratory illness, or acute otitis externa)
39	Antibiotics for adenoviral conjunctivitis	Do not order antibiotics for adenoviral conjunctivitis
40	Antidepressant monotherapy for bipolar disorder	Do not prescribe antidepressants as monotherapy for patients with bipolar I disorder
41^a^	Two or more concurrent antipsychotic medications	Do not routinely prescribe 2 or more antipsychotic medications concurrently

Abbreviations: ASA I or II, American Society of Anesthesiologists Physical Status Classification I or II; CAD, coronary artery disease; CKD, chronic kidney disease; CT, computed tomography; DEXA, dual-energy x-ray absorptiometry; ECG, electrocardiogram; EEG, electroencephalography; MRI, magnetic resonance imaging; PCI, percutaneous coronary intervention; PFT, pulmonary function testing; PSA, prostate-specific antigen.

^a^ Measures included in the main composite score. Measures with key numbers 8, 26, 29, 30, and 36 were not derived from the Milliman MedInsight Health Waste Calculator. Descriptions are adapted from Choosing Wisely and US Preventive Services Task Force recommendations.

Determining eligibility for each measure required analysis of data from the lookback (2015) and study (2016-2017) periods, including beneficiary age, sex, health care service receipt, medical diagnoses, and/or prescription receipt. For example, for the measure “injection for low back pain,” the measure denominator included eligible beneficiary-months after 2 or more diagnoses of low back pain (excluding radiculopathy) 7 or more days apart in 2016 and 2017. The measure numerator included eligible beneficiary-months for all beneficiaries in the denominator who also received a low-value injection (≥1 epidural, facet, or trigger point injection for a patient with a low back pain diagnosis and without etanercept on the same claim) on or after the second low back pain diagnosis date. We grouped measures into the following 5 clinical categories: laboratory testing, imaging, cardiopulmonary and neurologic testing, procedures, and drugs. See the eMethods and eTables 1 and 2 in Supplement 1 for measure details.

### Organizational, Attributed Beneficiary, and Area-Level Characteristics of Health Systems

We defined the following health system characteristics: size (number of physicians; IQVIA), specialty mix (proportion of all physicians specializing in primary care; IQVIA), insurance product ownership (system offers any insurance product; AHRQ Compendium), Accountable Care Organization (ACO) status (system participates in ≥1 ACO contract; AHRQ Compendium), profit status (for profit or nonprofit, which included “not for profit” and “government”; AHRQ Compendium), and teaching status (includes ≥1 major teaching hospital; AHRQ Compendium). Attributed beneficiary characteristics included proportion with Medicaid-Medicare full dual enrollment and proportion of non-White race (Master Beneficiary Summary File); race was treated as a binary measure (White and non-White race). Area-level characteristics included US Census region (based on system headquarters), area-level standardized, risk–adjusted, per capita health care spending (mean of Hospital Referral Region [HRR]–level spending for system-attributed beneficiaries based on residential zip code; MedInsight), and area-level hospital market concentration (mean of HRR-level Herfindahl-Hirschman index for system-attributed beneficiaries based on residential zip code; MedInsight).

### Statistical Analysis

Statistical analysis was conducted from January 26 to July 15, 2021. We estimated low-value care use by health systems for each of the 41 measures and averaged across measures within each clinical category. We tested for correlation (Pearson) in individual health systems between and within these clinical categories. We then created a composite measure using the 28 most common measures among health systems (defined by ≥11 beneficiaries receiving the service in at least 50% of systems). The composite score is the mean of proportions for the 28 measures, standardized to reflect distance from the all-systems mean value. We mapped systems with color coding reflecting the composite score to explore geographical patterns associated with low-value care intensity.

In sensitivity analyses, we calculated the composite score in 3 additional ways. First, we repeated the main composite score method for all 41 measures. Second, to address the possibility that some health systems may not have the capability to provide certain low-value services such that the main composite measure may give these systems undue “credit” for avoiding these services, we reestimated the main composite score using 23 of the 28 measures that required no specialized capability (eg, cardiac catheterization). Third, we reestimated the main composite score excluding 3 measures for which lookback periods of more than 1 year would be ideal to determine measure eligibility but were not available in our data. We used Spearman rank correlation to compare these sensitivity composites with the main composite score.

To explore attribution and accountability for low-value care use, we used a National Provider Identifier–to–health system linkage file based on IQVIA to calculate the measure-specific and overall proportions of low-value service recipients for whom a National Provider Identifier on the service claim was associated with the system to which the beneficiary was attributed.

Finally, to examine health system characteristics associated with low-value care use, we used bivariate analyses to report mean composite scores for each characteristic level and then performed multivariable linear regression to estimate mean composite scores adjusted for the other characteristics.

We created claims measures and ran descriptive, validation, regression, and correlation analyses using SAS, version 9.4 (SAS Institute Inc) and Stata, version 16.1 (StataCorp LLC). To create the figures, we used Python in Google Collaboratory and Tableau Public. Reported *P* values were 2-sided, and *P* < .05 represented statistical significance.

## Results

Our analysis included 556 systems serving a total of 11 637 763 beneficiaries; system-attributed cohorts ranged from 250 to 379 949 beneficiaries (mean [SD], 20 931 [34 663] beneficiaries) (eTable 3 in Supplement 1). Most health systems were nonprofit (533 [96%]), and 200 (36%) included at least 1 major teaching hospital. Systems had a mean (SD) size of 598 (1233) physicians, with a mean (SD) of 31% (9%) classified as primary care. Across attributed beneficiary cohorts, the mean (SD) proportion of women was 58% (3%), and the mean (SD) proportion dually enrolled in Medicaid was 10% (10%). Of the 556 systems, 375 (67%) had at least 5% of their attributed beneficiaries in 2 or more HRRs (mean [SD] number of HRRs per system, 2.4 [1.4]).

Across health systems, the mean (SD) use of each of the 41 low-value services ranged from 0% (0.002%) to 41% (7%) of eligible beneficiaries (eTable 4 in Supplement 2). The most common low-value services were antibiotics for acute upper respiratory and ear infections (mean [SD] rate, 41% [7%] of eligible beneficiaries), preoperative laboratory testing (mean [SD] rate, 28% [4%] of eligible beneficiaries), prostate-specific antigen testing in men older than 70 years (mean [SD] rate, 27% [8%]), and use of antipsychotic medications in patients with dementia (mean [SD] rate, 23% [8%]). Overall, 36% of all study beneficiaries (4 152 961 of 11 637 763) received at least 1 of the 41 low-value services.

**Figure 1.**
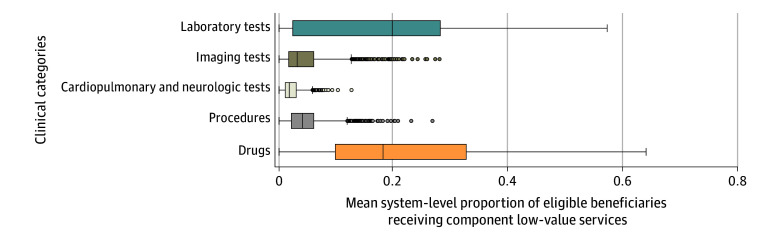
Distribution of Health System–Attributed Beneficiaries’ Use of Low-Value Services by Clinical Category Each box plot represents the distribution of the 556 system-level averages of the proportions of eligible attributed beneficiaries who received low-value services in the given clinical category. All 41 measures are subsumed under these clinical categories. The ends of the boxes represent the 25th and 75th percentile values; middle lines, median values; whiskers, minimum and maximum values within 1.5 times the interquartile range (IQR) of the median; dots, values more than 1.5 times the IQR from the median.

The most commonly used low-value clinical categories were laboratory testing and prescription drugs (Figure 1). Examining correlations between these clinical categories within health systems, we found significant positive correlations between 6 of 10 pairs (*r* = 0.14-0.46). The highest correlations were between laboratory testing and procedures (*r* = 0.46) and between laboratory testing and cardiopulmonary and neurologic testing (*r* = 0.43). Exploring within-category service correlation, we found significant correlations between 10 of 15 pairs of laboratory test measures (all positive), 28 of 66 pairs of imaging test measures (19 positive and 9 negative), 8 of 15 pairs of cardiopulmonary and neurologic testing measures (7 positive and 1 negative), 18 of 55 pairs of procedures (17 positive and 1 negative), and 5 of 15 pairs of drug measures (3 positive and 2 negative) (eTable 5 in Supplement 1).

### Low-Value Care Composite Measure

Based on the composite measure of 28 services, health systems with the lowest use of low-value care were Grady Health System (Georgia), Cambridge Health Alliance (Massachusetts), and North Memorial Health Care (Minnesota). Health systems with the greatest relative use of low-value services were El Camino Hospital (California), Huntington Memorial Hospital (California), and Eisenhower Medical Center (California) (eTable 4 in Supplement 2).

**Figure 2.**
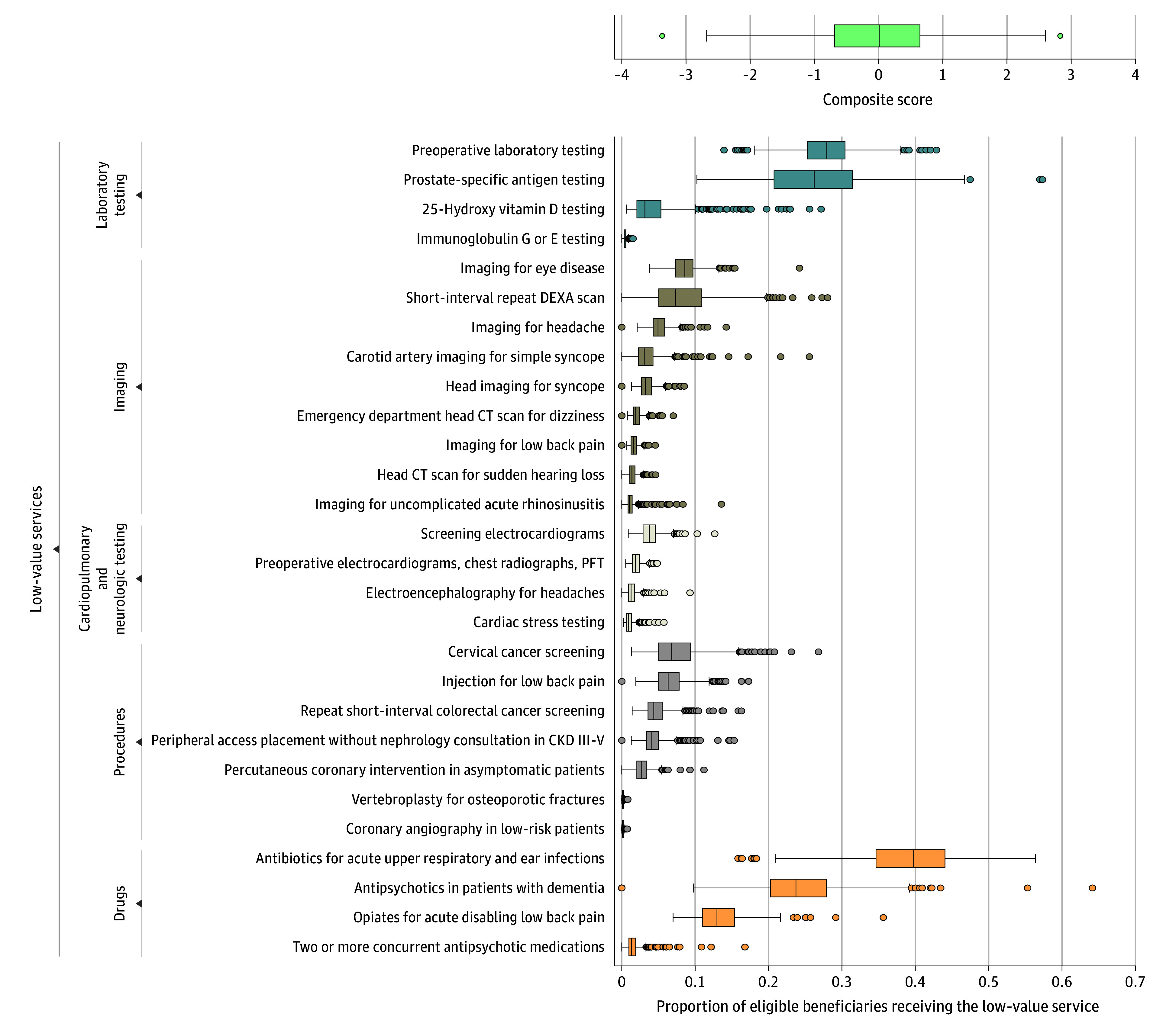
Distribution of Health System–Attributed Beneficiaries’ Use of the 28 Most Commonly Observed Low-Value Services Box plots reflect the distribution of composite scores (top) and distributions of use of individual low-value services (bottom) for eligible beneficiaries attributed to the 556 studied health systems. The composite score is the mean of proportions for the 28 measures, converted to a standardized score to measure distance from mean. Composite scores range from −3.39 to 2.83. Services displayed are the 28 most common of 41 measured (each was observed in at least 11 beneficiaries in at least 50% of systems studied). The ends of the boxes represent the 25th and 75th percentile values; middle lines, median values; whiskers, minimum and maximum values within 1.5 times the interquartile range (IQR) of the median; dots, values more than 1.5 times the IQR from the median. CKD III-V indicates chronic kidney disease stage III to stage V; CT, computed tomography; DEXA, dual-energy x-ray absorptiometry, and PFT, pulmonary function testing.

**Figure 3.**
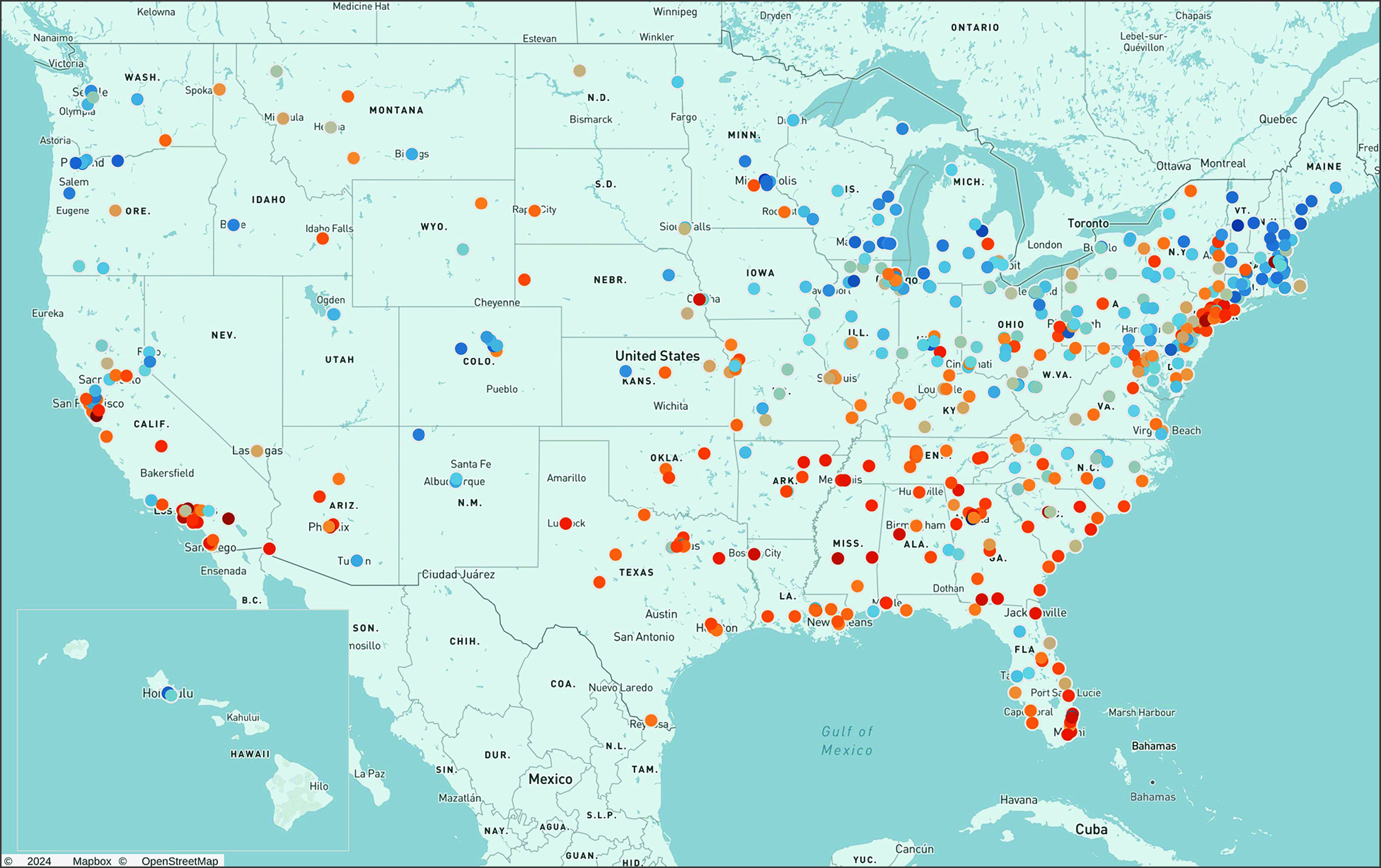
Map of the 556 Health System Headquarters and Associated System Composite Scores All of the studied health system headquarters are displayed, with their associated system composite score indicated by dot color. Blue denotes lower relative use of low-value care, and orange indicates higher relative use; increasing darkness indicates greater distance from the mean composite score of zero.

Figure 2 displays the distribution of system-level low-value service use for the 28 included measures. Figure 3 displays all studied health system headquarters on a map with their composite scores indicated by dot color. This map shows that beneficiaries attributed to health systems with headquarters in the Northeast and Midwest tended to receive fewer of the measured low-value services, while beneficiaries attributed to those in the South and certain US cities (eg, Los Angeles and New York) tended to receive more low-value services.

Sensitivity analyses using alternative approaches to composite score calculation generated very similar results (eResults in Supplement 1). A mean of 78% of patients receiving a given low-value service received it from a physician linked to their health system (eTable 6 in Supplement 1). This mean value varied by service, ranging from 35% for imaging for eye disease to 84% for vitamin D testing; for all but the eye imaging measure, the low-value service was ordered by an in-system physician for most recipients.

### Health System Characteristics and Low-Value Care

In bivariate analyses, health systems differed in low-value care use based on size, specialty mix, ACO status, teaching status, percentage of non-White–attributed beneficiaries, region, and area-level per capita health care spending (Table 2). In multivariable analysis, characteristics associated with more low-value care use were fewer primary care physicians (adjusted composite score, 0.14 [95% CI, 0.03-0.25] for systems with less than the median percentage of primary care physicians vs −0.15 [95% CI, –0.26 to –0.04] for those with more than the median percentage of primary care physicians; *P* < .001), no major teaching hospital (adjusted composite, 0.11 [95% CI, 0.00 to 0.22] without a teaching hospital vs −0.21 [95% CI, –0.37 to –0.05] with a teaching hospital; *P* = .003), headquartered in the South or West (adjusted composite, 0.27 [95% CI, 0.13-0.42] for the South and 0.15 [95% CI, –0.05 to 0.36] for the West compared with −0.09 [95% CI, –0.26 to 0.08] for the Northeast and −0.39 [95% CI, –0.54 to –0.23] for the Midwest; *P* < .001), and serving areas with more health care spending (adjusted composite, 0.25 [95% CI, 0.14-0.37] for areas above the median level of spending vs −0.26 [95% CI, –0.38 to –0.14] for areas below the median level of spending; *P* < .001).

**Table 2.** Health System Organizational, Attributed Beneficiary, and Area-Level Characteristics Associated With Low-Value Care Use^a^

Characteristic	Composite, mean (SD)	*P* value	Adjusted	
			Composite, mean (95% CI)	*P* value
Organizational				
Health system size (No. of physicians), quartile				
Lowest^b^	0.03 (1.0)	.01	−0.06 (−0.24 to 0.12)	.42
Middle 2	0.09 (1.06)		0.05 (−0.06 to 0.16)	
Top	−0.22 (0.84)		−0.05 (−0.24 to 0.14)	
Specialty mix^c^				
Below median	0.11 (1.04)	.01	0.14 (0.03 to 0.25)	<.001
Above median	−0.11 (0.95)		−0.15 (−0.26 to −0.04)	
Insurance product ownership				
Does not own insurance product	0.03 (1.05)	.37	−0.02 (−0.12 to 0.09)	.73
Owns insurance product	−0.05 (0.92)		0.01 (−0.11 to 0.14)	
ACO status				
Does not have an ACO	0.13 (1.01)	.01	0.04 (−0.09 to 0.16)	.41
Has an ACO	−0.1 (0.98)		−0.03 (−0.14 to 0.07)	
Profit status				
Nonprofit	−0.02 (1.01)	.1	−0.01 (−0.08 to 0.07)	.59
For profit	0.37 (0.68)		0.11 (−0.3 to 0.51)	
Teaching status				
Does not include major teaching hospital	0.09 (1.03)	.003	0.11 (0.0 to 0.22)	.003
Includes major teaching hospital	−0.17 (0.92)		−0.21 (−0.37 to −0.05)	
Attributed beneficiary				
Medicaid-Medicare dual enrollment, %				
≤20%	0.02 (0.98)	.15	0.02 (−0.07 to 0.1)	.17
>20%	−0.19 (1.18)		−0.2 (−0.49 to 0.09)	
Non-White race, %^d^				
≤20%	−0.09 (0.94)	.001	−0.05 (−0.15 to 0.04)	.08
>20%	0.22 (1.11)		0.13 (−0.04 to 0.29)	
Area-level				
Census region of system headquarters				
Northeast	−0.26 (1.03)	<.001	−0.09 (−0.26 to 0.08)	<.001
South	0.49 (0.84)		0.27 (0.13 to 0.42)	
Midwest	−0.38 (0.8)		−0.39 (−0.54 to −0.23)	
West	−0.01 (1.14)		0.15 (−0.05 to 0.36)	
Standardized risk-adjusted per capita health care spending, $^e^				
Below median	−0.3 (1.02)	<.001	−0.26 (−0.38 to −0.14)	<.001
Above median	0.3 (0.90)		0.25 (0.14 to 0.37)	
Hospital market concentration (HHI)^f^				
Below median	−0.01 (1.05)	.76	−0.01 (−0.12 to 0.1)	.86
Above median	0.01 (0.95)		0.0 (−0.1 to 0.11)	

Abbreviations: ACO, accountable care organization; HHI, Herfindahl-Hirschman Index.

^a^ Beneficiary characteristics are sourced from the Master Beneficiary Summary File and US 2010 Census. System characteristics are based on IQVIA OneKey 2016 and the Agency for Healthcare Research and Quality 2016 Compendium of US Health Systems. Standardized health care spending and hospital market concentration were obtained from MedInsight.

^b^ Health system quartile cutoffs: lowest, less than 100 physicians; middle, 100 to 637 physicians; and highest, 638 physicians or more.

^c^ Specialty mix cutoff: below median, less than 31.5% primary care physicians; above median, 31.5% or more primary care physicians.

^d^ Non-White race defined as those without non-Hispanic White race and ethnicity.

^e^ Standardized prices per capita cutoffs: below median, less than $9415.04; above median, $9415.04 or more.

^f^ The HHI is an economic measure of market concentration; higher numbers indicate a more concentrated market (ie, a market served by fewer hospitals).

## Discussion

Building on past measurements of low-value care at the national and regional levels, we measured low-value care at the actionable level of individual health systems.^1,19,24^ Absolute values suggest the level of potential waste, while comparison reveals the broad range of low-value care use; health systems in the lowest range of low-value care use demonstrate what can be achieved. The identification of health system characteristics (such as specialty mix) that are associated with low-value care use suggest a focus for future research on the causes of and remedy for overuse.

Our results highlight potential contributors to system-level patterns of low-value care. Laboratory testing and prescription drugs were the most frequently used low-value services, which is not surprising given the broad base of clinicians ordering these services.^25^ This finding corroborates prior work and validates inclusion of these services in many Choosing Wisely lists and as targets for intervention.^1,26^ We found a substantial number of positive, significant within-system correlations between and within clinical measure groups, consistent with prior work showing modestly positive associations between service categories within Veterans Affairs medical centers and within provider organizations (ie, organizational units ranging from individual physicians to health systems).^27,28^ Correlations between cardiac screening measures and between head imaging measures raise the possibility that system factors, such as screening protocols or capital investment in imaging technology, may contribute to system-specific practice patterns. At the same time, some heterogeneity in low-value care provision within health systems was not surprising given the diverse drivers of these services and is consistent with prior studies showing limited correlations between quality measures.^29,30,31^ Each health system may perform well in some aspects of low-value care and poorly in others based on numerous factors that create unique low-value care “fingerprints.”

Health system characteristics associated with low-value care use echo prior work and suggest directions for future research. We found that having more primary care physicians was associated with less low-value care use, building on the work by Zhou et al^24^ showing that each additional primary care physician per 1000 residents in HRRs was associated with a substantial decrease in a low-value care composite of 20 measures and evidence that primary care physicians account for a small share of low-value spending, as well as studies linking primary care with lower overall health care spending.^32,33^ We did not find a significant association between having an ACO contract and low-value care use, similar to prior research showing only modest reductions.^21^ A 2012-2015 survey of ACO executives found that just 32% of ACOs had implemented strategies to reduce the use of low-value services.^34^ We found that health systems without a major teaching hospital used more low-value care, consistent with work by Chalmers et al^35^ comparing hospital-specific low-value services between teaching and nonteaching hospitals. We found that health systems with greater use of low-value care were clustered in the South and West, consistent with extensive work on low-value care.^19,35,36^ Systems in higher-spending areas provided more low-value care, on average, mirroring research demonstrating a correlation between regional low-value care spending and total spending.^19^ This finding also highlights the relationship between large systems and regional practice pattern measures that reflect the sum of their constituents.

Health system organizational features are likely key drivers of low-value care through policy setting, workflows and protocols, culture, and compensation models.^27^ Our focus on system-level measurement is particularly important because an increasing number of individuals in the US receive care from system-affiliated clinicians each year,^37^ and we found that most beneficiaries receiving these low-value services received them from health system physicians. This finding could have both positive and negative implications for efforts to curb low-value care.^38^ On the positive side, health systems can leverage efficiencies of scale to apply quality metrics across all affiliated clinicians; track performance outcomes; streamline and disseminate new evidence, programs, or technologies to improve care delivery (eg, clinical decision support tools and evidence-based guidelines); and foster a culture of health care resource stewardship.^39,40^ On the negative side, even though hospitals are financially disincentivized to perform low-value inpatient services by the diagnosis-related group payment system, large health systems that offer many hospital-based procedures have higher commercial prices and incentives to refer patients to other system-affiliated clinicians and services. This situation may result in increases in marginal services,^41,42,43^ such as low-value magnetic resonance imaging studies, as a recent study demonstrated among physicians newly employed by health systems.^43^

### Limitations

This study has important limitations. Although we use widely vetted claims-based definitions of low-value care, claims data do not contain the nuanced clinical details necessary for more precise value measurement and risk misclassifying appropriate services as low-value. To mitigate this risk, we used the most specific service definitions available. Known variation in the quality and quantity of physician coding practices may influence measures that rely on diagnostic codes.^44^ If systems differ systematically in the completeness of diagnoses recorded, this may bias our conclusions. Although we expect that the low-value care patterns observed among large cohorts of fee-for-service Medicare enrollees likely reflect practice patterns of the systems studied, subpopulations as well as practices and hospitals within each system may have distinct care patterns. We limited our study to 556 large health systems that collectively served about 40% of all fee-for-service Medicare beneficiaries aged 65 years or older in our study period, but our findings may not be generalizable to other systems or independent practices. Because we limited the main composite measure to the 28 most common services to reduce the influence of rare events, its interpretation is limited to the scope of measures included. These measures represent a narrow share of all low-value care and an even smaller share of care quality overall. The composite score is most valuable at the extremes of the range: identifying relative outliers. Finally, all results represent care patterns in our study period and may not reflect current practice.

## Conclusions

This system-level assessment of recent low-value practice patterns may enable cross-system comparisons and empower a range of stakeholders with actionable metrics. Research has shown that multicomponent interventions are most effective at reducing low-value care.^45^ For policy makers and payers, future repeated measurements may demonstrate how systems are changing over time and may reveal interventions associated with the increase or decrease of low-value services. Health systems might use this report to inform the internal measurement of low-value care. If internal measurements confirm our findings, they could illuminate factors associated with specific low-value services and inform strategies to address them. For example, because laboratory testing is among the most frequently used low-value services and is a common component of standard order sets, health system leaders could revise such tools to ensure that they promote high-value care. In addition, state or regional quality improvement organizations could use such results to establish a health system learning community focused on reducing the most frequently used low-value services in that region.^46^

Health systems could use system-level data on low-value care to develop incentive schemes to reduce unnecessary care, educate their workforce, or link hiring and retention decisions to use of low-value services. Health plans might include specific targets for the reduction of low-value care in contract negotiations or create networks of “high-value” clinicians. System-level reporting may also help to inform patients about specific low-value services and where they may be at greater risk of receiving them. Future measurement might emphasize other important facets of low-value care, such as cost, potential for direct harm, and potential for care cascades.^36,47^ Even with its imperfections, the transparent and actionable measurement of low-value care is a critical step toward improving the quality and affordability of US health care.

## Notes

Supplement 1. **eMethods.** Milliman MedInsight Waste Calculator Measures **eTable 1.** Measure Definitions **eTable 2.** Selected Milliman MedInsight Health Waste Calculator Specifications **eTable 3.** Health System Organizational, Attributed Beneficiary, and Area-Level Characteristics **eTable 5.** Correlations Between and Within Clinical Categories **eResults.** Sensitivity Analyses **eTable 6.** Number of Eligible Beneficiaries and Proportion of Eligible Beneficiaries Whose Attributed Health System Matches the System Associated With NPI on the Low Value Service Claim (by Measure)

Supplement 2. **eTable 4.** Low Value Service Use by Health Systems: by Individual Service Type and Overall
