# Supplementary material for: Low-Value Care at the Actionable Level of Individual Health Systems
Source: JAMA Intern Med. 2021 Sep 27;181(11):1490–500. doi: 10.1001/jamainternmed.2021.5531 (PMC8477305; doi:10.1001/jamainternmed.2021.5531)
Supplement: Supplement 1. — eMethods. Milliman MedInsight Waste Calculator Measures eTable 1. Measure Definitions eTable 2. Selected Milliman MedInsight Health Waste Calculator Specifications eTable 3. Health System Organizational, Attributed Beneficiary, and Area-Level Characteristics eTable 5. Correlations Between and Within Clinical Categories eResults. Sensitivity Analyses eTable 6. Number of Eligible Beneficiaries and Proportion of Eligible Beneficiaries Whose Attributed Health System Matches the System Associated With NPI on the Low Value Service Claim (by Measure) [file jamainternmed-e215531-s001.pdf]

Supplementary Online Content

Ganguli I, Morden NE, Yang CWW, Crawford M, Colla CH. Low-value care at the actionable level of individual health systems. *JAMA Intern Med.* Published online September 27, 2021. doi:10.1001/jamainternmed.2021.5531

eMethods. Milliman MedInsight Waste Calculator Measures
eTable 1. Measure Definitions
eTable 2. Selected Milliman MedInsight Health Waste Calculator Specifications
eTable 3. Health System Organizational, Attributed Beneficiary, and Area-Level Characteristics
eTable 5. Correlations Between and Within Clinical Categories
eResults. Sensitivity Analyses
eTable 6. Number of Eligible Beneficiaries and Proportion of Eligible Beneficiaries Whose Attributed Health System Matches the System Associated With NPI on the Low-Value Service Claim (by Measure)

This supplementary material has been provided by the authors to give readers additional information about their work.

© 2021 Ganguli I et al. *JAMA Internal Medicine*.

eMethods. Milliman MedInsight Waste Calculator Measures

Beneficiaries were included in measure-specific denominators based on diagnosis or other inclusion criteria for each specific wasteful service. This was done using diagnosis codes extracted from the “Starting Population” field of the Milliman software’s technical documentation, *Waste Clinical Collections_v7.0*, from 2016-2017. See https://medinsight.com/healthcare-data-analytics-software/analytic-products/clinical-suite/health-waste-calculator/

Beneficiaries were included in measure-specific numerators if they had any claim flagged by the Calculator as Degree of Certainty=”W” (Wasteful) and with the Sufficient History flag=”Y” for the specific measure (except three measures noted in the Appendix table footnotes, for which the maximum lookback period exceeded 1 year; for these, the sufficient history requirement was waived.)

Wasteful services in 2015 were not counted in the measure proportions, though available 2015 data were fed into the calculator only to provide lookback information.

Milliman Waste Calculator-based measures were computed using beneficiary-level weights to reflect time contributed in 2016-2017.

All measures accounted for the 2015 ICD-9 to ICD-10 transition.

© 2021 Ganguli I et al. *JAMA Internal Medicine*.

eTable 1. Measure Definitions

Key Number	Measure	Denominator	Numerator	Eligible beneficiary-months	Calculator-based
1	Preoperative laboratory testing	Study beneficiaries with low-risk surgery (WCC_SET "Low Risk Surgery"); limited to 40% sample and ptD enrollment for all AB months.	All study beneficiaries in denominator with >=1 claim flagged by Calculator as "Wasteful" and Sufficient History flag="Y" for MEASURE_ID ASA01a	Months in 2016-17 with parts A, B, and D enrollment	Yes
2	Prostate-specific antigen testing (PSA) testing*	All male study beneficiaries over 70 years of age	All study beneficiaries in denominator with >=1 claim flagged by Calculator as "Wasteful" for MEASURE_ID URG01	Months in 2016-17 with parts A and B enrollment	Yes
3	25-hydroxy vitamin D testing	All study beneficiaries; limited to 40% sample and ptD enrollment for all AB months.	All study beneficiaries in denominator with >=1 claim flagged by Calculator as "Wasteful" and Sufficient History flag="Y" for MEASURE_ID SCP01	Months in 2016-17 with parts A, B, and D enrollment	Yes
4	Testing for chronic urticaria	All study beneficiaries w/ Diagnosis of Urticaria (WCC_SET: "Urticaria")	All study beneficiaries in denominator with >=1 claim flagged by Calculator as "Wasteful" and Sufficient History flag="Y" for MEASURE_ID AI03	Months in 2016-17 with parts A and B enrollment	Yes
5	Immunoglobulin G or E testing	All study beneficiaries w/ diagnosis of Allergy (WCC_Set: "Allergies")	All study beneficiaries in denominator with >=1 claim flagged by Calculator as "Wasteful" and Sufficient History flag="Y" for MEASURE_ID AI02	Months in 2016-17 with parts A and B enrollment	Yes
6	Bleeding time testing	All study beneficiaries	All study beneficiaries in denominator with >=1 claim flagged by Calculator as "Wasteful" and Sufficient History flag="Y" for MEASURE_ID SCP05	Months in 2016-17 with parts A and B enrollment	Yes
7	Imaging for eye disease	All study beneficiaries	All study beneficiaries in denominator with >=1 claim flagged by Calculator as "Wasteful" and Sufficient History flag="Y" for MEASURE_ID AO02	Months in 2016-17 with parts A and B enrollment	Yes

© 2021 Ganguli I et al. *JAMA Internal Medicine*.

8	Short-interval repeat dual-energy x-ray absorptiometry (DEXA) scan	Female with at least one dual energy X-ray absorptiometry (DEXA) in 2017. Exclude beneficiary if they have fragility fracture in 2015-2017 on or prior to date of last cohort-defining DEXA, if they have a diagnosis of cancer in 2015-2017, or if they are not PartA/Part B or are covered by a Health Maintenance Organization or located in the US or >65 at end of the year in both 2015 and 2016.	All study beneficiaries in denominator with >=1 claim of DEXA within >30 to <730 days before a cohort-defining DEXA	None	No
9	Imaging for headache	All study beneficiaries with diagnosis of headache (WCC_Set: "Headache")	All study beneficiaries in denominator with >=1 claim flagged by Calculator as "Wasteful" and Sufficient History flag="Y" for MEASURE_ID ACR01	Months in 2016-17 with parts A and B enrollment	Yes
10	Carotid artery imaging for simple syncope	All study beneficiaries with diagnosis of Syncope (WCC_Set: "Syncope")	All study beneficiaries in denominator with >=1 claim flagged by Calculator as "Wasteful" and Sufficient History flag="Y" for MEASURE_ID AN02	Months in 2016-17 with parts A and B enrollment	Yes
11	Head imaging for syncope	All study beneficiaries with diagnosis of Syncope (WCC_Set: "Syncope")	All study beneficiaries in denominator with >=1 claim flagged by Calculator as "Wasteful" and Sufficient History flag="Y" for MEASURE_ID ACPY01	Months in 2016-17 with parts A and B enrollment	Yes
12	Emergency department head computed tomography (CT) scan for dizziness	All study beneficiaries with diagnosis from WCC_Set "Dizziness" w/ emergency/urgent care visit type	All study beneficiaries in denominator with >=1 claim flagged by Calculator as "Wasteful" and Sufficient History flag="Y" for MEASURE_ID JH001	Months in 2016-17 with parts A and B enrollment	Yes
13	Imaging for low back pain	Study beneficiaries with diagnosis of low back pain (WCC_Set: "Low Back Pain"); limited to 40% sample w/ ptD enrollment for all AB months.	All study beneficiaries in denominator with >=1 claim flagged by Calculator as "Wasteful" and Sufficient History flag="Y" for MEASURE_ID AFP02	Months in 2016-17 with parts A, B, and D enrollment	Yes
14	Head computed tomography (CT) scan for sudden hearing loss	All study beneficiaries with diagnosis of sudden hearing loss (WCC_Set: "Sudden Hearing Loss")	All study beneficiaries in denominator with >=1 claim flagged by Calculator as "Wasteful" and Sufficient History flag="Y" for MEASURE_ID AOHN01	Months in 2016-17 with parts A and B enrollment	Yes
15	Imaging for uncomplicated acute rhinosinusitis	All study beneficiaries with diagnosis of sudden acute	All study beneficiaries in denominator with >=1 claim flagged by Calculator as	Months in 2016-17 with parts A and B enrollment	Yes

© 2021 Ganguli I et al. *JAMA Internal Medicine*.

		rhinosinusitis (WCC_Set: "Acute Rhinosinusitis")	"Wasteful" and Sufficient History flag="Y" for MEASURE_ID AOHN04		
16	Magnetic resonance imaging (MRI) for rheumatoid arthritis	All study beneficiaries with diagnosis of rheumatoid arthritis (WCC_Set: "Rheumatoid Arthritis")	All study beneficiaries in denominator with >=1 claim flagged by Calculator as "Wasteful" and Sufficient History flag="Y" for MEASURE_ID ACRH03	Months in 2016-17 with parts A and B enrollment	Yes
17	Coronary artery calcium scoring for known coronary artery disease (CAD)	All study beneficiaries with diagnosis of CAD (WCC_Set: "CAD")	All study beneficiaries in denominator with >=1 claim flagged by Calculator as "Wasteful" and Sufficient History flag="Y" for MEASURE_ID SCCT01	Months in 2016-17 with parts A and B enrollment	Yes
18	Dual-energy X-ray absorptiometry (DEXA) scan in low-risk patients	All male study beneficiaries age 66-70 at end of year.	All study beneficiaries in denominator with >=1 claim flagged by Calculator as "Wasteful" and Sufficient History flag="Y" for MEASURE_ID AFP03	Months in 2016-17 with parts A and B enrollment	Yes
19	Screening electrocardiograms (ECGs)*	All study beneficiaries	All study beneficiaries in denominator with >=1 claim flagged by Calculator as "Wasteful" for MEASURE_ID AFP05	Months in 2016-17 with parts A and B enrollment	Yes
20	Preoperative electrocardiograms (ECG), chest radiographs, or pulmonary function testing (PFT)	All study beneficiaries with low risk surgery (WCC_set: "Low-Risk Surgery")	All study beneficiaries in denominator with >=1 claim flagged by Calculator as "Wasteful" and Sufficient History flag="Y" for MEASURE_ID ASA01b	Months in 2016-17 with parts A and B enrollment	Yes
21	Electroencephalography (EEG) for headaches	All study beneficiaries with diagnosis of headache (WCC_Set: "Headache")	All study beneficiaries in denominator with >=1 claim flagged by Calculator as "Wasteful" and Sufficient History flag="Y" for MEASURE_ID ASA01b	Months in 2016-17 with parts A and B enrollment	Yes
22	Cardiac stress testing	All study beneficiaries	All study beneficiaries in denominator with >=1 claim flagged by Calculator as "Wasteful" and Sufficient History flag="Y" for MEASURE_ID ACC00	Months in 2016-17 with parts A and B enrollment	Yes
23	Pulmonary function testing (PFT) prior to cardiac surgery	All study beneficiaries with cardiac surgery (WCC_Set: "Cardiac Surgery")	All study beneficiaries in denominator with >=1 claim flagged by Calculator as "Wasteful" and Sufficient History flag="Y" for MEASURE_ID STHS05	Months in 2016-17 with parts A and B enrollment	Yes
24	Preoperative echocardiography or cardiac stress testing	All study beneficiaries with low or moderate risk surgery (WCC_Set: "Low or Moderate Risk Surgery"	All study beneficiaries in denominator with >=1 claim flagged by Calculator as "Wasteful" and Sufficient History flag="Y" for MEASURE_ID ASA02	Months in 2016-17 with parts A and B enrollment	Yes
25	Cervical cancer screening*	All female study beneficiaries	All study beneficiaries in denominator with >=1 claim flagged by Calculator as	Months in 2016-17 with parts A and B enrollment	Yes

© 2021 Ganguli I et al. *JAMA Internal Medicine*.

			"Wasteful" for MEASURE_ID AFP00		
26	Injection for low back pain	Beneficiaries with 2 diagnoses of low back pain >=7 days apart. Exclude beneficiary if they have any diagnosis of radiculopathy in 2016-2017	All study beneficiaries in denominator with >=1 epidural, facet, trigger point injection claim for a diagnosis of low back pain without etanercept on the same claim. Injection claim must be on or after the second diagnosis of low back pain.	Months in 2016-2017 with parts A and B enrollment after 2nd diagnosis of low back pain	No
27	Repeat short-interval colorectal cancer screening	All study beneficiaries	All study beneficiaries in denominator with >=1 claim flagged by Calculator as "Wasteful" and Sufficient History flag="Y" for MEASURE_ID GE01	Months in 2016-17 with parts A and B enrollment	Yes
28	Peripheral access placement without nephrology consultation in stage III-V chronic kidney disease (CKD)	All study beneficiaries with diagnosis of stage III-V chronic kidney disease (WCC_Set: "Stage III-V CKD"	All study beneficiaries in denominator with >=1 claim flagged by Calculator as "Wasteful" and Sufficient History flag="Y" for MEASURE_ID SNP01	Months in 2016-17 with parts A and B enrollment	Yes
29	Feeding tubes for patients with dementia	Study beneficiaries with >=2 diagnoses of dementia >=7 days apart and is a long-term nursing home resident (combined length of stay in nursing facility >90 days across 2016-2017)	All study beneficiaries in denominator with >=1 procedure code for feeding tube on claim after both second diagnosis of dementia and first day of institutionalization	Months in 2016-2017 with parts A and B enrollment after both second diagnosis of dementia and first day of institutionalization	No
30	Percutaneous coronary intervention (PCI) for asymptomatic patients	Study beneficiaries with ischemic heart disease (defined by presence of Chronic Conditions Warehouse first indication date prior to December 31 of 2017) and established diagnosis of acute myocardial infarction (defined by presence of CCW first indication date prior to July 1, 2017)	All study beneficiaries in denominator with percutaneous coronary intervention (coronary stent, balloon angioplasty, or atherectomy) >=6 months after CCW 1st indication date for both acute myocardial infarction and ischemic heart disease. Exclude beneficiary if the PCI is during or within 14 days after an emergency department visit.	Months in 2016-2017 with parts A and B enrollment after both first indication dates of acute myocardial infarction and ischemic heart disease.	No
31	Vertebroplasty for osteoporotic fractures	All study beneficiaries	All study beneficiaries in denominator with >=1 claim flagged by Calculator as "Wasteful" and Sufficient History flag="Y" for MEASURE_ID DOR121	Months in 2016-17 with parts A and B enrollment	Yes
32	Coronary angiography in low-risk patients	All study beneficiaries	All study beneficiaries in denominator with >=1 claim flagged by Calculator as "Wasteful" and Sufficient History flag="Y" for MEASURE_ID SNUC01	Months in 2016-17 with parts A and B enrollment	Yes

© 2021 Ganguli I et al. *JAMA Internal Medicine*.

33	Multiple palliative radiaotherapy treatments for bone metastases	All study beneficiaries with diagnosis of bone metastases (WCC_Set: "Bone Metastases")	All study beneficiaries in denominator with >=1 claim flagged by Calculator as "Wasteful" and Sufficient History flag="Y" for MEASURE_ID HPM03	Months in 2016-17 with parts A and B enrollment	Yes
34	Renal artery revascularization	All study beneficiaries	All study beneficiaries in denominator with >=1 claim flagged by Calculator as "Wasteful" and Sufficient History flag="Y" for MEASURE_ID DOR124	Months in 2016-17 with parts A and B enrollment	Yes
35	Arthroscopic lavage and debridement for knee osteoarthritis	All study beneficiaries with diagnosis of knee osteoarthritis (WCC_set: "Knee Osteoarthritis")	All study beneficiaries in denominator with >=1 claim flagged by Calculator as "Wasteful" and Sufficient History flag="Y" for MEASURE_ID DOR21	Months in 2016-17 with parts A and B enrollment	Yes
36	Antipsychotics for patients with dementia	Study beneficiaries with >=2 diagnoses of dementia >=7 days apart anytime in 2016-2017. Exclude if beneficiary has had any diagnosis of severe mental illness in the same period. Limited to 40% sample	All study beneficiaries with any antipsychotic prescription with a fill date after second dementia diagnosis.	Months of part D coverage after second diagnosis of dementia	No
37	Opiates for acute disabling low back pain	Study beneficiaries with diagnosis of low back pain (WCC_Set: "Low Back Pain"); limited to 40% sample and any months ptD enrollment.	All study beneficiaries in denominator with >=1 claim flagged by Calculator as "Wasteful" and Sufficient History flag="Y" for MEASURE_ID AAPMR05	Months in 2016-17 with parts A, B, and D enrollment.	Yes
38	Antibiotics for acute upper respiratory tract and ear infections	Study beneficiaries with diagnosis of upper respiratory infection or ear infection (WCC_Set: "URI or Ear Infection"); limited to 40% sample and any months ptD enrollment.	All study beneficiaries in denominator with >=1 claim flagged by Calculator as "Wasteful" and Sufficient History flag="Y" for MEASURE_ID AP00	Months in 2016-17 with parts A, B, and D enrollment	Yes
39	Antibiotics for adenoviral conjunctivitis	Study beneficiaries with diagnosis of adenoviral conjunctivitis (WCC_Set: "Adenoviral Conjunctivitis"); limited to 40% sample and any months ptD enrollment	All study beneficiaries in denominator with >=1 claim flagged by Calculator as "Wasteful" and Sufficient History flag="Y" for MEASURE_ID AO03	Months in 2016-17 with parts A, B, and D enrollment	Yes

© 2021 Ganguli I et al. *JAMA Internal Medicine*.

40	Antidepressant monotherapy for bipolar disorder	Study beneficiaries with diagnosis of bipolar disorder (WCC_Set: "Bipolar Disorder"); limited to 40% sample and ptD enrollment for all AB months.	All study beneficiaries in denominator with >=1 claim flagged by Calculator as "Wasteful" and Sufficient History flag="Y" for MEASURE_ID DOR85	Months in 2016-17 with parts A, B, and D enrollment	Yes
41	Two or more concurrent antipsychotic medications	Study beneficiaries with any prescription fill for an antipsychotic (WCC_Set: "Antipsychotics"); limited to 40% sample w/ ptD enrollment for all AB months	All study beneficiaries in denominator with >=1 claim flagged by Calculator as "Wasteful" and Sufficient History flag="Y" for MEASURE_ID APA01	Months in 2016-17 with parts A, B, and D enrollment	Yes

*Because the maximum lookback period specified for this measure exceeded 1 year, the "Sufficient History" flag requirement was waived for this measure. All beneficiaries are over age 65 during study period, which may affect the eligible population for some measures.

© 2021 Ganguli I et al. *JAMA Internal Medicine*.

eTable 2. Selected Milliman MedInsight Health Waste Calculator Specifications

MEASURE ID & NAME	STARTING POPULATION	EXCLUSION	NOT WASTEFUL	LIKELY WASTEFUL	WASTEFUL	Last Updated	CITATIONS
AAPMR05 - Opioids for Acute Back Pain*	All members aged 18 years and older with a prescription of opioids within 28 days of a diagnosis of low back pain.	None.	Members with a diagnosis of cancer or sickle cell anemia 180 days prior to the opioid prescription.	Members with a diagnosis of low back pain and a prescription for NSAIDs or tramadol or duloxetine within 90 days on or prior to the index event (visit for a diagnosis of back pain).	All remaining.	Feb-18	http://annals.org/aim/fullarticle/2603228/noninvasive-treatments-acute-subacute-chronic-low-back-pain-clinical-practice
ACC00 - Cardiac stress testing*	All members aged 18 years and older with a service for cardiac stress testing.	Members from the starting population with:<br>· An inpatient admissions 30 days prior to the cardiac stress testing; or<br>- Emergency care or observation care within 1 day on or after the cardiac stress testing; or<br>· Coronary angiography on the day of the cardiac stress testing; or<br>· PCI/CABG within 30 days on or after the cardiac stress testing.	Members with a service for:<br>- Stress testing (stress EKG, cardiac radionuclide imaging, and stress echo) and a diagnosis of cardiac symptoms or ventricular tachycardia within 90 days prior to the cardiac stress testing; or<br>· Stress CMR and a diagnosis of ventricular tachycardia within 90 days prior to the cardiac stress testing; or<br>· Advanced stress testing (cardiac radionuclide imaging, stress echo or stress CMR) and a diagnosis of cardiac conditions within 90 days prior to the cardiac stress testing; or<br>· Stress EKG with cardiac rehabilitation and a diagnosis of heart failure within 90 days prior to the cardiac stress testing; or<br>· Stress echocardiography and a diagnosis of valve disease or cardiomyopathy within 1 year prior to the cardiac stress testing; or<br>· Kidney or liver transplant and a diagnosis of pre-operative cardiovascular examination within 30 days prior to the pre-operative cardiac stress testing.	· Members with stress EKG and a diagnosis of cardiac conditions (heart failure, ventricular fibrillation, abnormal EKG findings, and coronary stenosis) within 90 days prior to the cardiac stress testing; or<br>· Member aged more than 40 years with 2 or more different risk factors (diabetes mellitus, hypertension, hyperlipidemia, obesity, coronary artery disease, peripheral artery disease) on the day of the stress testing.	All remaining.	Feb-18	http://annals.org/aim/article/1363528/screening-coronary-heart-disease-electrocardiography-u-s-preventive-services-task

© 2021 Ganguli I et al. *JAMA Internal Medicine*.

MEASURE ID & NAME	STARTING POPULATION	EXCLUSION	NOT WASTEFUL	LIKELY WASTEFUL	WASTEFUL	Last Updated	CITATIONS
ACPY01 - Brain Imaging Studies (CT or MRI) for Simple Syncope*	All members aged 18 years and older with a brain imaging study (CT or MRI) within 30 days of a diagnosis of syncope.	Members from the starting population with:<br>· An inpatient admission within the time period from the diagnosis of syncope to the brain imaging; or<br>· A competing diagnosis (CVA, intracranial hemorrhage, brain tumors etc.) within 90 days prior to the brain imaging; or<br>· Head Injury within 7 days prior to the brain imaging; or<br>· Diagnosis of benign or malignant tumors of the head and neck within 1 year prior to the brain imaging.	Members with a diagnosis of neurological deficits within the time period from the diagnosis of syncope to the brain imaging.	None.	All remaining.	Feb-18	http://www.acr.org/~/media/1C1F7C7A570D469A9C411D95067BDF94.pdf
ACR01 - Imaging for Uncomplicated Headache*	All members aged 18 years and older with a diagnosis of uncomplicated headache within 30 days prior to a head imaging.	Members from the starting population with:<br>- An inpatient admission within the time period from the diagnosis of headache to the head imaging; or<br>- Diagnosis of cancer or head trauma within 1 year prior to the head imaging; or<br>- Diagnosis of complicated sinusitis/mastoiditis/middle ear disease within 180 days prior to the head imaging.	All members with:<br>- Head MRI/ MRA AND Age >55 years AND raised ESR/temporal arteritis within the service unit; or<br>- CT/MRI/CT/MRA and diagnosis of complicated headache within the service unit; or<br>- Members with a diagnosis of underlying conditions (post traumatic headache, neurologic deficit, epilepsy, ataxia) who obtained a service for MRI/CT within the service unit; or<br>- MRI/CT and diagnosis of underlying conditions (Trigeminal headache, immunocompromised conditions) within the service unit; or<br>- MRI/CT and diagnosis of pregnancy without diagnosis of headache in the last 270 days prior to the index event; or<br>- MRI and diagnosis of meningitis/encephalitis or chronic conditions within the service unit; or<br>- MRI/MRA/CT and diagnosis of cerebrovascular event within the service unit.	All members with:<br>- CT/CTA and Age >55 years and raised ESR/temporal arteritis within the service unit; or<br>- CT/MRA/CTA and diagnosis of chronic conditions (Trigeminal headache immunocompromised conditions) within the service unit; or<br>- MRA/CTA and diagnosis of underlying conditions (post traumatic headache, neurologic deficit) within the service unit; or<br>- CT head and diagnosis of meningitis/encephalitis within the service unit; or<br>- Diagnosis of chronic headache within 1 year prior to the MRI head imaging.	All remaining	Feb-18	https://www.aafp.org/afp/2013/0515/p682.pdf

© 2021 Ganguli I et al. *JAMA Internal Medicine*.

MEASURE ID & NAME	STARTING POPULATION	EXCLUSION	NOT WASTEFUL	LIKELY WASTEFUL	WASTEFUL	Last Updated	CITATIONS
ACRH03 – MRI for Rheumatoid Arthritis*	All members aged 18 years and older with a diagnosis of rheumatoid arthritis (with preceding RA diagnosis within 365 days) who obtained an MRI study within 90 days of RA diagnosis.	None	None	None	All starting population	Mar 2016	http://ard.bmj.com/content/annrheumdis/72/6/804.full.pdf
AFP00 - Cervical Cancer Screening in Women*	All female members with a cervical cancer screening testing service.	Members from the starting population with a diagnosis of HIV as far back in claims data prior to the cervical cancer screening testing.	· Members aged 21 years and older with a diagnosis of high risk conditions for developing cervical cancer or with gynecologic malignancy or dysplasia as far back in claims data; or<br>· Members aged 21 years or older and any documented abnormal Pap smear findings within 3 years prior to the cervical testing; or<br>· Members aged between 21 and 64 years who had cervical cytology once in 3 years and no codes of total hysterectomy as far back in claims data; or<br>· Members aged between 30 and 64 years who had cervical cytology and HPV testing on the same day and once in 5 years and had no codes of total hysterectomy as far back in claims data .	Members aged 21 years or older and a diagnosis of potential cervical cancer risk conditions (inflammatory disease of cervix uteri, co-infection with herpes simplex etc.) within 14 days prior to the cervical cancer screening testing.	All remaining.	Feb-18	http://www.uspreventiveservicestaskforce.org/Page/Document/RecommendationStatementFinal/cervical-cancer-screening

© 2021 Ganguli I et al. *JAMA Internal Medicine*.

MEASURE ID & NAME	STARTING POPULATION	EXCLUSION	NOT WASTEFUL	LIKELY WASTEFUL	WASTEFUL	Last Updated	CITATIONS
AFP02 - Imaging for Low Back Pain*	All members 18 years of age and older with a low back imaging service and diagnosis of low back pain within 6 weeks prior to the low back imaging.	Members from the starting population with a:<br>· Diagnosis of low back pain within 180 days prior to back imaging; or<br>· Lumbar spine surgery within 90 days prior to back Imaging; or<br>· Inpatient admission within 6 weeks prior to the back Imaging.	· Back MRI with a diagnosis of neurological deficits within the service unit; or<br>·MRI with diagnosis of other serious underlying conditions (cancer, immunosuppression) as far back prior to the back MRI; or<br>· Diagnosis of conditions requiring imaging (osteoporosis, trauma, drug abuse, infection) within 90 days of back MRI; or<br>· Age 70 years and older with back X-ray/CT lumbar spine without contrast/MRI; or<br>· X-ray/CT lumbar spine without contrast/MRI and long term steroid use; or<br>· Diagnosis of conditions requiring imaging (osteoporosis, trauma etc.) within 90 days of X-ray/CT lumbar spine without contrast.	Members with:<br>- Diagnosis of other serious underlying conditions (cancer, immunosuppression) and X-ray/CT lumbar spine as far back possible prior to the back imaging; or<br>· Diagnosis of any neurological deficits and a CT lumbar spine within the service unit.	All remaining.	Feb-18	https://acsearch.acr.org/docs/69483/Narrative/
AFP03 - DEXA Screening for Osteoporosis *	All women under 65 years of age and men 50-69 years of age who had a DEXA screening with an office visit within 30 days prior to the DEXA screening.	Members from the starting population with a diagnosis of osteoporosis on the day of the DEXA or as far back in claims data prior to the DEXA screening.	Members with:<br>- A diagnosis of major risk factors for developing osteoporosis (vertebral compression fracture, malabsorption syndrome, osteopenia etc.) within 2 years prior to the DEXA screening; or<br>· At least two potential risk factors for developing osteoporosis (rheumatoid arthritis, hyperthyroidism etc.) within 2 years prior to the DEXA screening.	None.	All remaining.	Feb-18	http://annals.org/aim/article/746858/screening-osteoporosis-u-s-preventive-services-task-force-recommendation-statement

© 2021 Ganguli I et al. *JAMA Internal Medicine*.

MEASURE ID & NAME	STARTING POPULATION	EXCLUSION	NOT WASTEFUL	LIKELY WASTEFUL	WASTEFUL	Last Updated	CITATIONS
AFP05 - Annual EKGs or Cardiac Screening in Asymptomatic Population*	All members aged 18 years and older with a service for EKG or any other cardiac screening with an emergency/outpatient/inpatient visit within 14 days prior to the EKG or cardiac screening service.	Members from the starting population with:<br>- Diagnosis of inflammatory conditions such as arthritis, joint pains, myositis etc. within 14 days prior to the EKGs or other cardiac screening; or<br>- Low risk surgery within 30 days after the EKGs or other cardiac screening; or<br>- Inpatient stay on or 30 days prior to the EKGs or other cardiac screening.	Members with:<br>- Diagnosis of high risk markers for CHD (Diabetes, atherosclerotic disease etc.) within 2 years prior to the EKGs or other cardiac screening; or<br>- Diagnosis of two or more risk factors suggestive of intermediate CHD risk (Obesity, family history of ischemic heart disease etc.) within 2 years prior to the EKGs or other cardiac screening; or<br>- Diagnosis of two or more signs or symptoms suggestive of CHD (Chest pain, atrial flutter, tachycardia etc.) within 60 days on or prior to the EKGs or other cardiac screening.	None.	All remaining.	May-19	https://www.uspreventiveservicestaskforce.org/Page/Document/RecommendationStatementFinal/coronary-heart-disease-screening-with-electrocardiography
AI02 - Immunoglobulin G (IgG) or Immunoglobulin E (IgE) Tests in the Evaluation of Allergy*	All members with IgG or IgE testing and had a diagnosis of allergy within 30 days prior to the IgG or IgE testing.	None.	Members with IgE testing and:<br>- A diagnosis of eczema or dermatographism within 1 year prior to the IgE allergy test, or<br>· Children less than 15 years old.	· Members with a diagnosis of migraine and food allergy within 14 days prior to IgG allergy test; or<br>· Members with a diagnosis of atopic allergy within 14 days prior to the IgE allergy test.	All remaining.	Feb-18	http://www.aaaai.org/Aaaai/media/MediaLibrary/PDF%20Documents/Practice%20and%20Parameters/allergydiagnostictesting.pdf
AI03 - Diagnostic Testing for Chronic Urticaria*	All members with routine diagnostic testing and a diagnosis of chronic urticaria within 365 days prior to the diagnostic testing.	Members from the starting population with:<br>- A single allergy diagnostic testing service or where the count of the allergy test is more than one but without a diagnosis of urticaria.	None.	None.	All remaining.	Feb-18	https://www.aaaai.org/Aaaai/media/MediaLibrary/PDF%20Documents/Practice%20and%20Parameters/Urticaria-2014.pdf
AN01 - Electroencephalography for Headaches*	All members with an EEG and a diagnosis of headache within 30 days prior to the EEG.	Members from the starting population with:<br>- Inpatient admission in between the diagnosis of headache and a service for EEG.	Members with:<br>- Diagnosis of epilepsy or seizures within 180 days prior to EEG; or<br>- Diagnosis of abnormal involuntary movement between the diagnosis of headache and a service for EEG.	None.	All remaining.	Mar-16	http://staywell.com/wp-content/uploads/2013/12/Headache0113.pdf

© 2021 Ganguli I et al. *JAMA Internal Medicine*.

MEASURE ID & NAME	STARTING POPULATION	EXCLUSION	NOT WASTEFUL	LIKELY WASTEFUL	WASTEFUL	Last Updated	CITATIONS
AN02 - Imaging of the Carotid Arteries for Simple Syncope*	All members aged 18 years and older with a carotid duplex ultrasound imaging and a diagnosis of syncope within 14 days prior to the carotid artery duplex ultrasound.	Members from the starting population with:<br>- An inpatient admission between the diagnosis of simple syncope and a service of carotid duplex ultrasound.	Members with:<br>- Diagnosis of neurological deficit in between simple syncope and a service of carotid duplex ultrasound.	None.	All remaining.	Feb-18	https://www.ncbi.nlm.nih.gov/pmc/articles/PMC3295536/
AO02 - Imaging Tests for Eye Disease	All members who obtained an eye imaging test	None	· Members with conditions requiring posterior optical coherence tomography with ophthalmologist visit who obtained posterior optical coherence tomography within 10 days<br>· Members with conditions requiring anterior optical coherence tomography with ophthalmologist visit who obtained anterior optical coherence tomography within 10 days<br>· Members with conditions requiring fundus photography with ophthalmologist visit who obtained fundus photography within 10 days<br>· Members with conditions requiring visual field testing with ophthalmologist visit who obtained visual field testing within 10 days<br>· Members with conditions requiring external eye photography with ophthalmologist visit who obtained external eye photography within 10 days<br>· Members with conditions requiring internal eye photography with ophthalmologist visit who obtained internal eye photography within 10 days	None	All remaining.	Feb 2018	https://www.aao.org/preferred-practice-pattern/comprehensive-adult-medical-eye-evaluation-2015

© 2021 Ganguli I et al. *JAMA Internal Medicine*.

MEASURE ID & NAME	STARTING POPULATION	EXCLUSION	NOT WASTEFUL	LIKELY WASTEFUL	WASTEFUL	Last Updated	CITATIONS
AO03 - Antibiotics Prescription for Adenoviral Conjunctivitis*	All members with antibiotics prescription and a diagnosis of adenoviral conjunctivitis within 14 days prior to the antibiotics prescription.	None.	Members with:<br>- Diagnosis of secondary bacterial infection identified by associated otitis media or with symptoms of mucopurulent discharge from the eye within 14 days prior to the antibiotic prescription; or<br>- Diagnosis of skin conditions (such as impetigo, ecthyma, infected eczemas, cellulitis, erysipelas, infected wounds, MRSA infections etc.,) within 14 days prior to the antibiotic prescription.	None.	All remaining.	Feb-18	https://www.aoa.org/documents/CPG-11.pdf
AOHN01 - CT Head/Brain for Sudden Onset Hearing Loss*	All members with a CT scan of the head/brain within 7 days of sudden hearing loss.	Members from the starting population with:<br>- Competing diagnosis (meningitis, hemiplegia, subarachnoid hemorrhage) within 30 days on or prior to CT of head or brain.	Members with:<br>- Diagnosis of acoustic neuroma/CVA within 90 days on or prior to the CT of head or brain and contraindications to MRI (pacemakers, metallic implants, severe claustrophobia, etc.) as far back as possible in member's history; or<br>- Diagnosis of pregnancy anytime between CT and diagnosis of sudden hearing loss; or<br>- Diagnosis of history of trauma involving the ear within 3 days prior to the CT of head or brain; or<br>- History of chronic ear disease or other related disease (Paget disease, fibrous dysplasia, encephalopathy or bone metastasis to the temporal bone, benign or malignant tumors of the petrous temporal bone, etc.) within 1 year prior to the CT of head or brain.	None.	All remaining.	Feb-18	http://journals.sagepub.com/doi/pdf/10.1177/0194599812436449

© 2021 Ganguli I et al. *JAMA Internal Medicine*.

MEASURE ID & NAME	STARTING POPULATION	EXCLUSION	NOT WASTEFUL	LIKELY WASTEFUL	WASTEFUL	Last Updated	CITATIONS
AOHN04 - Imaging for Uncomplicated Acute Rhinosinusitis*	All members with sinus imaging within 30 days of acute rhinosinusitis.	Members from the starting population with:<br>- Inpatient admissions within 30 days prior to imaging; or<br>- Competing diagnosis (headache, hearing loss, syncope and dizziness/vertigo) within 30 days prior to the imaging.	Members with:<br>- Diagnosis of chronic sinusitis within 30 days prior to imaging for sinus or head CT; or<br>- Diagnosis of acute or recurrent sinusitis on 3 different from dates within one year prior to imaging for sinus or head CT; or<br>- Diagnosis of complicated rhinosinusitis within 180 days prior to CT/MRI imaging; or<br>- Diagnosis of complicated rhinosinusitis (orbital or intracranial complications with ocular or neurologic deficits, preseptal or post septal cellulitis, sub periosteal abscess, orbital cellulitis or abscess) within 180 days prior to sinus CT/MRI.	Members with:<br>- Diagnosis of immunodeficiency or acute frontal or sphenoidal sinusitis within 180 days prior to sinus CT.	All remaining.	Feb-18	https://www.aafp.org/afp/2016/0715/p97.pdf
AP00 - Antibiotics for acute upper respiratory and ear infections*	All members aged 3 months and older with a prescription of oral antibiotics within 7 days of upper URI or ear infection (acute sinusitis, URI, viral respiratory illness or acute otitis externa).	Members from the starting population with:<br>- Diagnosis for comorbid conditions (HIV, malignant neoplasms or immunocompromised conditions etc.) within 1 year prior to the prescription of antibiotics; or<br>- Competing diagnosis (acute URI or acute external otitis in the presence of competing diagnosis (abscess, cellulitis, acute infections and other infectious conditions) within 30 days prior to the prescription of antibiotics; or<br>- Tympanostomy tube placement up to 2 years prior to prescription of antibiotics; or<br>- Diagnosis of otitis media within 14 days prior to the prescription of antibiotics.	Members with:<br>- Diagnosis for acute rhinosinusitis and sinusitis complications within 10 days prior to the antibiotic prescription; or<br>- Diagnosis of acute otitis externa and middle ear disease within 30 days prior to the antibiotic prescription; or<br>- Diagnosis for malignant otitis externa within 30 days prior to the antibiotic prescription.	None.	All remaining.	Jan-17	http://annals.org/aim/article/2481815/appropriate-antibiotic-use-acute-respiratory-tract-infection-adults-advice-high

© 2021 Ganguli I et al. *JAMA Internal Medicine*.

MEASURE ID & NAME	STARTING POPULATION	EXCLUSION	NOT WASTEFUL	LIKELY WASTEFUL	WASTEFUL	Last Updated	CITATIONS
APA01 - Concurrent Use of Two or More Antipsychotic Medications*	All members who were prescribed antipsychotics.	Members from the starting population with:<br>- Prescription for lithium within 60 days prior to antipsychotic prescription.	Members without any concurrent prescription of 2 different antipsychotic medication within 30 days consecutively for a period of 60 days prior antipsychotic prescription.	None.	All remaining.	Nov-19	http://www.choosingwisely.org/clinician-lists/american-psychiatric-association-routine-prescription-of-two-or-more-concurrent-antipsychotics/
ASA01a - Preoperative Baseline Laboratory Studies*	All members aged 2 years or older with a baseline laboratory testing within 30 days prior to a low-risk non-cardiac surgery.	Members from the starting population with:<br>- E&M visit for emergency care, observation or urgent care within one day prior to the Pre-operative testing; or<br>- Electrolyte testing laboratory related services and prescription of medications such as digoxin, diuretics and angiotensin converting enzyme inhibitors or angiotensin receptor blockers within 90 days prior to the prior Pre-operative testing; or<br>- Diagnosis of endocrine, liver or renal disorders within 180 days prior to the prior Pre-operative testing; or<br>- Diagnosis of history of anemia or history suggestive of recent blood loss within 180 days prior to the Pre-operative CBC testing; or<br>- Coagulation testing related services and a diagnosis of coagulation disorders within 180 days prior to the Pre-operative CBC testing; or<br>- Prescription of anticoagulant medications within 90 days on or prior to coagulation testing related services.	Members with:<br>- Urinalysis prior to urologic procedure or after the diagnosis of urinary symptoms or disorders.	None.	All remaining.	May-19	http://anesthesiology.pubs.asahq.org/article.aspx?articleid=2443414

© 2021 Ganguli I et al. *JAMA Internal Medicine*.

MEASURE ID & NAME	STARTING POPULATION	EXCLUSION	NOT WASTEFUL	LIKELY WASTEFUL	WASTEFUL	Last Updated	CITATIONS
ASA01b - Preoperative EKG, Chest X-Ray and Pulmonary Function Testing*	All members aged 2 years or older with an EKG, chest X-ray and pulmonary function within 30 days prior to low-risk surgery.	Members from the starting population with:<br>- E&M visit for emergency care, observation or urgent care on or within one day prior to the Pre-operative testing.	Members with:<br>- Diagnosis of cardiovascular risk factors within 90 days prior to the Pre-operative testing; or<br>-Signs and symptoms of cardiovascular and cardiopulmonary disease within 30 days prior to the pre-operative testing; or<br>- Diagnosis of underlying pulmonary disease within 90 days prior to the Pre-operative testing.	None.	All remaining.	May-19	http://anesthesiology.pubs.asahq.org/article.aspx?articleid=2443414
ASA02 - Preoperative Cardiac Echocardiography or Stress Testing*	All members aged 18 years or older with an echocardiography or stress testing within 30 days prior to a low or intermediate risk non-cardiac surgery.	Members from the starting population with:<br>- Inpatient admission on or 30 days prior to the Pre-operative echocardiography or stress testing; or<br>- E&M visit for emergency care, observation or urgent care on or within one day prior to Pre-operative echocardiography or stress testing; or<br>- Diagnosis of high risk markers for CHD within 2 years prior to Pre-operative echocardiography or stress testing.	Members with:<br>- Diagnosis of two or more different signs or symptoms suggestive of CHD within 60 days prior to Pre-operative echocardiography or stress testing.	None.	All remaining.	May-19	http://circ.ahajournals.org/content/130/24/2215.long
DOR121 – Vertebroplasty	All members aged 18 years and older with a service for vertebroplasty	None	Members with a diagnosis of spinal or vertebral conditions (hemangioma of spine, multiple myeloma, eosinophilic granuloma or Kummell Disease) within 1 year on or prior to vertebroplasty service.	None	All remaining.	May-17	http://onlinelibrary.wiley.com/doi/10.1002/14651858.CD006349.pub2/epdf

© 2021 Ganguli I et al. *JAMA Internal Medicine*.

MEASURE ID & NAME	STARTING POPULATION	EXCLUSION	NOT WASTEFUL	LIKELY WASTEFUL	WASTEFUL	Last Updated	CITATIONS
DOR124 - Renal Artery Revascularization*	All members with renal artery revascularization.	None.	Members with:<br>- Diagnosis of fibromuscular dysplasia within 90 days prior to renal artery revascularization.	Members with:<br>- Diagnosis of malignant hypertension within 90 days prior to renal artery revascularization; or<br>- Diagnosis of chronic kidney disease stage III to stage VI within 90 days prior to renal artery revascularization; or<br>- Diagnosis of pulmonary edema or acute coronary syndrome within 90 days prior to renal artery revascularization.	All remaining.	Feb-18	http://www.nejm.org/doi/full/10.1056/NEJMoa0905368
DOR21 - Arthroscopic Lavage and Debridement for Knee Osteoarthritis*	All members aged 18 years and older with arthroscopic lavage or debridement within 365 days of knee osteoarthritis.	None.	None.	None.	All remaining.	Feb-18	http://www.aaos.org/research/guidelines/TreatmentofOsteoarthritisoftheKneeGuideline.pdf
DOR85 - Antidepressants Monotherapy in Bipolar Disorder*	All members with a prescription of antidepressants within 3 days of bipolar disorder.	None.	Members with:<br>- Prescription for mood stabilizers within 90 days prior to antidepressants monotherapy.	None.	All remaining.	Feb-18	https://www.healthquality.va.gov/bipolar/bd_306_sum.pdf
GE01 - Colorectal Cancer Screening in Adults 50 Years and Older*	All members aged 50 years and older with colorectal cancer screening.	Members from the starting population with:<br>- Diagnosis of colorectal cancer, family or personal history of colorectal cancer or colon adenoma as far back as possible in members' history; or<br>- Service for total colectomy as far back as possible in members' history.	Members with:<br>- FOBT once in a year; or<br>- Immunochemical-based fecal occult blood testing once in a year; or<br>- FIT-DNA once in a year; or<br>- Flexible sigmoidoscopy once in 5 years; or<br>- CT colonography once in 5 years; or<br>- Colonoscopy once in 10 years.	None.	All remaining.	Feb-18	http://www.cancer.org/acs/groups/cid/documents/webcontent/003170-pdf.pdf

© 2021 Ganguli I et al. *JAMA Internal Medicine*.

MEASURE ID & NAME	STARTING POPULATION	EXCLUSION	NOT WASTEFUL	LIKELY WASTEFUL	WASTEFUL	Last Updated	CITATIONS
HPM03 – Multiple Palliative Radiation Treatments in Bone Metastases	All members with a diagnosis of bone metastasis who had external radiation therapy performed	Members with prior radiation therapy and bone metastases on the same day more than 14 days prior to the external radiation therapy	· Diagnosis of complications of bone metastases<br>· Members without external radiation treatment within 14 days prior to radiation therapy	None	All remaining.	Feb-18	http://www.redjournal.org/article/S0360-3016(10)03577-7/pdf
JH001 - CT Scans for Emergency Room Evaluation of Dizziness*	All members aged 18 years and older with CT scan within 1 day of dizziness.	Members from the starting population with:<br>- Diagnosis of Comorbid conditions (headache or hearing loss or complicated sinusitis/mastoiditis/middle ear disorder) within 30 days prior to CT scan; or<br>- Inpatient admission within the service unit.	Members with:<br>- Competing diagnosis (a history of recent head injury or other brain conditions) within 7 days prior to CT scan; or<br>- Diagnosis of benign or malignant tumors of the head and neck within one year prior to CT scan.	None.	All remaining.	Feb-18	https://www.ncbi.nlm.nih.gov/pmc/articles/PMC2676794/pdf/nihms102245.pdf
SCCT01 - Coronary Artery Calcium Scoring for Known CAD*	All members aged 18 years and older with calcium scoring within 1 year of coronary artery disease.	None.	None.	None.	All starting population.	Feb-18	http://circ.ahajournals.org/content/129/25_suppl_2/S49.long
SCP01 - Screening for Vitamin D Deficiency*	All members with vitamin-D testing (25-OH-Vitamin D and 1, 25-dihydroxyvitamin D testing).	None.	Members with:<br>- Diagnosis of chronic conditions within one year prior to 25-OH-vitamin D testing; or<br>- Diagnosis of risk factors within 90 days prior to 25-OH-vitamin D testing; or<br>- Prescription for high risk medications within 90 days prior to 25-OH-vitamin D testing; or<br>- Diagnosis of pregnancy and obesity on the day of 25-OH-vitamin D testing; or<br>- Age 65 years or older with any history of falls or a history of non-traumatic fractures within 1 year prior to 25-OH-vitamin D testing; or<br>- Diagnosis of inherited or acquired disorders of vitamin D and phosphate metabolism within 90 days prior to 1,25 (OH)2 -vitamin D testing.	None.	All remaining.	Feb-18	http://annals.org/aim/fullarticle/1938935/screening-vitamin-d-deficiency-adults-u-s-preventive-services-task

© 2021 Ganguli I et al. *JAMA Internal Medicine*.

MEASURE ID & NAME	STARTING POPULATION	EXCLUSION	NOT WASTEFUL	LIKELY WASTEFUL	WASTEFUL	Last Updated	CITATIONS
SCP05 - Bleeding Time Testing*	All members with a bleeding test.	None.	None.	None.	All starting population.	Feb-18	http://www.ajmc.com/journals/issue/2010/2010-09-vol16-n09/ajmc_10sep_wu_xcl_e220to227
SNP01 - Peripherally Inserted Central Catheters in Stage III-V CKD Patients*	All members with PICC placement within 1 year of stage III-V Chronic Kidney disease (III-V).	None.	Members with:<br>- Nephrology consult within 7 days prior to the PICC line insertion.	None.	All remaining.	May-19	http://c.ymcdn.com/sites/www.asdin.org/resource/resmgr/imported/ASDINVeinPreservation.pdf
SNUC01 - Coronary Angiography*	All members aged 18 years or older with coronary angiography.	Members from the starting population with:<br>- Cardiac transplant status or congenital cardiac anomalies as far back as possible in members' history.	Members with:<br>- Cardiac conditions (acute coronary syndrome, myocardial infarction, heart failure, or ventricular fibrillation or ventricular tachycardia) within 30 days prior to coronary angiography; or<br>- A service for cardiac valve surgeries within 30 days prior to coronary angiography<br>- Diagnosis of Obstructive coronary artery disease or PCI/CABG as far back with symptoms of chronic heart disease or abnormal cardiovascular study results within 30 days prior to coronary angiography; or<br>- A service for stress test and symptoms of chronic heart disease and abnormal cardiovascular study results within 14 days prior to coronary angiography.	Members with:<br>- Diagnosis of other cardiac conditions (atrial fibrillation, heart block, left bundle branch block, valve disease, cardiomyopathies, pericardial disease and stenosis/ regurgitation) on the day of Coronary angiography; or<br>-Diagnosis of pre-operative cardiovascular examination and 3 or more different high risk conditions (ischemic heart disease, diabetes mellitus, renal insufficiency, CVA on the day of coronary angiography; or<br>- A service for stress test with symptoms of chronic heart disease or abnormal cardiovascular study results within 14 days prior to coronary angiography.	All remaining.	Feb-18	http://www.sciencedirect.com/science/article/pii/S0735109713061470?via%3Dihub
STHS05 - Pulmonary Function Testing Before Cardiac Surgery*	All members aged 18 years and older with pulmonary function testing within 30 days prior to cardiac surgery.	None.	Members with a diagnosis of:<br>- Any underlying pulmonary disease within 90 days prior to pulmonary function testing; or<br>- Respiratory symptoms within 30 days prior to pulmonary function testing.	None.	All remaining.	Feb-18	https://jamanetwork.com/journals/jama/fullarticle/2510916

© 2021 Ganguli I et al. *JAMA Internal Medicine*.

MEASURE ID & NAME	STARTING POPULATION	EXCLUSION	NOT WASTEFUL	LIKELY WASTEFUL	WASTEFUL	Last Updated	CITATIONS
URG01 – Prostate-Specific Antigen (PSA) Screening for Prostate Cancer	All men who obtained a PSA-based screening test for prostate cancer	None	Members with a diagnosis of prostate cancer or who have a risk of recurrence of prostate cancer (up to 5 years prior to PSA test)	Members who have clinical presentations and risk factors for prostate cancer for suspected prostate cancer (up to 5 years prior to PSA test)	All remaining.	May-17	http://annals.org/aim/article/1216568/screening-prostate-cancer-u-s-preventive-services-task-force-recommendation

From Mafi et al. Supplement E table 1 [Trends in Low-Value Health Service Use and Spending in the US Medicare Fee-for-Service Program, 2014-2018. Mafi JN, Reid RO, Baseman LH, Hickey S, Totten M, Agniel D, Fendrick AM, Sarkisian C, Damberg CL. JAMA Netw Open. 2021 Feb 1;4(2):e2037328. doi: 10.1001/jamanetworkopen.2020.37328.] as provided by Dr. Leena Laloo of Milliman. Those not marked with "" were extracted independently by the study team using Milliman documentation.

© 2021 Ganguli I et al. *JAMA Internal Medicine*.

eTable 3. Health System Organizational, Attributed Beneficiary, and Area-Level Characteristics

	Total systems (N=556)
Organizational	
Health system size (number of physicians), mean (SD)	598 (1,233)
Specialty mix, (physicians in primary care), % mean (SD)	30.7 (9.4)
Owns insurance product, N (%)	218[a] (59.4)
Accountable Care Organization status, N (%)	315 (56.7)
Non-profit status, N (%)	533[a] (95.9%)
Teaching hospital status (system includes at least one major teaching hospital), N (%)	200[a] (36.0%)
Attributed beneficiary	
Systems total attributed cohort (min 250, max 379,949), mean (SD)	20,931 (34,663)
Age, mean (SD)	76.5 (1.0)
Female, % mean (SD)	57.6 (2.7)
Medicaid-Medicare dual enrollment, % mean (SD)	10.4 (10.0)
Race and ethnicity, % mean (SD)	
Non-Hispanic White	83.2 (15.7)
Black	8.0 (10.7)
Hispanic	4.4 (7.5)
Asian	2.1 (4.5)
Area-level	
Region of system headquarters, N (%)	
Northeast	138 (24.8%)
South	187 (33.6%)
Midwest	141 (25.4%)
West	90 (16.2%)
Standardized risk-adjusted per capita healthcare spending, $ mean (SD)	9328.11 (746.55)
Hospital market concentration (HHI),[b] mean (SD)	153.6 (316.0)

A total of 11,637,763 beneficiaries were attributed to 556 systems**.** For certain attributed cohort variables (total attributed and age) and area-level variables based on beneficiaries (standardized risk-adjusted per capital healthcare spending and hospital market concentration), we present means of system-level means; for sex and race, we provide means of system-level proportions, such that percentages do not add to 100%. Beneficiary characteristics are sourced from the Master Beneficiary Summary File and US 2010 Census. System characteristics are based on IQVIA 2016 and the Agency for Healthcare Research and Quality 2016 Compendium. Standardized per capita healthcare spending and hospital market concentration were obtained from Milliman MedInsight.

[a] Missing information on 19 systems for insurance product ownership, 1 system for profit status, and 1 system for teaching status; percentages reported for available data. Due to the Centers for Medicare and Medicaid Services suppression rules, we can only report rates of low-value service use if we do not report the specific number of attributed beneficiaries for each system; hence, we have reported this information as ranges.

[b] Herfindahl–Hirschman Index, or HHI, is an economic measure of market concentration; higher numbers indicate a more concentrated market (i.e., a market served by fewer hospitals).

© 2021 Ganguli I et al. *JAMA Internal Medicine*.

eTable 5. Correlations Between and Within Clinical Categories

5a. Correlations between clinical categories

	Laboratory Testing	Imaging	Cardiopulmonary and neurologic testing	Procedures	Drugs
Laboratory Testing	1				
Imaging	0.274*	1			
Cardiopulmonary and neurologic testing	0.431*	0.139*	1		
Procedures	0.457*	0.376*	0.195*	1	
Drugs	0.036	-0.031	-0.042	-0.014	1

*Represents P-values <0.05. Blue values are positive correlations, orange values are negative correlations.

5b. Correlations between measures within laboratory testing category

	Preoperative laboratory testing	Prostate-specific antigen (PSA) testing	25-hydroxy vitamin D testing	Immunoglobulin G or E testing	Testing for chronic urticaria	Bleeding time testing
Preoperative laboratory testing	1					
Prostate-specific antigen (PSA) testing	0.1534*	1				
25-hydroxy vitamin D testing	0.129*	0.0976*	1			
Immunoglobulin G or E testing	0.2074*	0.11*	0.243*	1		
Testing for chronic urticaria	0.0955*	0.0796	-0.0218	0.2605*	1	
Bleeding time testing	-0.0241	0.1567*	-0.0382	0.0004	0.1036*	1

*Represents P-values <0.05. Green denotes measures used in the main composite. Blue values are positive correlations, orange values are negative correlations.

© 2021 Ganguli I et al. *JAMA Internal Medicine*.

5c. Correlations between measures within imaging category

	Imaging for eye disease	Short-interval repeat dual-energy x-ray absorptiometry (DEXA) scan	Imaging for headache	Carotid artery imaging for simple syncope	Head imaging for syncope	Emergency department head computed tomography (CT) scan for dizziness	Imaging for low back pain	Head computed tomography (CT) scan for sudden hearing loss	Imaging for uncomplicated acute rhinosinusitis	Magnetic resonance imaging (MRI) for rheumatoid arthritis	Coronary artery calcium scoring for known coronary artery disease (CAD)	DEXA scan in low-risk patients
Imaging for eye disease	1											
Short-interval repeat dual-energy x-ray absorptiometry (DEXA) scan	0.1382*	1										
Imaging for headache	-0.0788	-0.0708	1									
Carotid artery imaging for simple syncope	-0.0028	0.1329*	0.0822	1								
Head imaging for syncope	-0.0743	-0.0667	0.3149*	0.0914*	1							
Emergency department head computed tomography (CT) scan for dizziness	-0.0897*	-0.1283*	0.3655*	-0.0653	0.3771*	1						
Imaging for low back pain	-0.1108*	-0.0881*	0.151*	0.1209*	0.213*	0.2055*	1					
Head computed tomography (CT) scan for sudden hearing loss	-0.0971*	-0.1938*	0.0927*	-0.0804	0.0877*	0.1009*	-0.14*	1				
Imaging for uncomplicated acute rhinosinusitis	-0.0802	-0.015	-0.0432	0.134*	0.0506	-0.0328	0.1915*	-0.0383	1			
Magnetic resonance imaging (MRI) for rheumatoid arthritis	0.1114*	0.2426*	-0.0569	-0.0255	-0.0178	-0.0641	-0.0257	-0.0965*	-0.0121	1		
Coronary artery calcium scoring for known coronary artery disease (CAD)	0.1635*	0.1653*	-0.024	-0.0271	0.0187	-0.0149	-0.0005	-0.1374*	-0.0446	0.0713	1	
Dual-energy X-ray absorptiometry (DEXA) scan in low-risk patients	0.0691	-0.0655	0.0295	-0.0563	-0.0066	0.077	-0.0429	0.0193	-0.0488	0.0046	0.0642	1

*Represents P-values <0.05. Green denotes measures used in the main composite. Blue values are positive correlations, orange values are negative correlations. DEXA=Dual-energy X-ray absorptiometry.

© 2021 Ganguli I et al. *JAMA Internal Medicine*.

5d. Correlations between measures within cardiopulmonary and neurologic testing category

	Preoperative electrocardiograms (ECG), chest radiographs, or pulmonary function testing (PFT)	Screening electrocardiograms (ECGs)	Electroencephalography (EEG) for headaches	Cardiac stress testing	Pulmonary function testing (PFT) prior to cardiac surgery	Preoperative echocardiography or cardiac stress testing
Preoperative electrocardiograms (ECG), chest radiographs, or pulmonary function testing (PFT)	1					
Screening electrocardiograms (ECGs)	0.4386*	1				
Electroencephalography (EEG) for headaches	-0.045	-0.0871*	1			
Cardiac stress testing	0.2386*	0.0217	0.1263*	1		
Pulmonary function testing (PFT) prior to cardiac surgery	0.0902*	0.0825	-0.0165	0.0429	1	
Preoperative echocardiography or cardiac stress testing	0.3038*	0.4054*	0.0024	0.2134*	0.0458	1

*Represents P-values <0.05. Green denotes measures used in the main composite. Blue values are positive correlations, orange values are negative correlations.

© 2021 Ganguli I et al. *JAMA Internal Medicine*.

5e. Correlations between measures within procedure category

	Cervical cancer screening	Injection for low back pain	Repeat short-interval colorectal cancer screening	Peripheral access placement without nephrology consultation in stage III-V chronic kidney disease (CKD)	Feeding tubes for patients with dementia	Percutaneous coronary intervention (PCI) for asymptomatic patients	Vertebroplasty for osteoporotic fractures	Coronary angiography in low-risk patients	Multiple palliative radiation treatments for bone metastases	Renal artery revascularization	Arthroscopic lavage and debridement for knee osteoarthritis
Cervical cancer screening	1										
Injection for low back pain	0.142*	1									
Repeat short-interval colorectal cancer screening	0.4167*	0.049	1								
Peripheral access placement without nephrology consultation in stage III-V chronic kidney disease (CKD)	0.0136	-0.036	-0.0235	1							
Feeding tubes for patients with dementia	0.225*	-0.0577	0.1558*	0.2878*	1						
Percutaneous coronary intervention (PCI) for asymptomatic patients	0.0369	0.0586	0.0603	-0.0311	-0.0556	1					
Vertebroplasty for osteoporotic fractures	0.0005	0.0706	0.089*	0.0923*	0.1066*	0.0046	1				
Coronary angiography in low-risk patients	0.0053	0.0186	0.0536	0.1675*	0.1619*	0.3583*	0.0322	1			
Multiple palliative radiation treatments for bone metastases	-0.0048	0.0055	0.0362	-0.0462	0.0209	-0.1381*	-0.0309	0.0098	1		
Renal artery revascularization	0.1069*	0.0401	0.0708	0.182*	0.2052*	0.142*	0.056	0.2174*	0.0226	1	
Arthroscopic lavage and debridement for knee osteoarthritis	0.0572	-0.0646	0.0456	0.0497	0.0574	0.0583	0.1908*	0.0089	-0.062	0.1018*	1

*Represents P-values <0.05. Green denotes measures used in the main composite. Blue values are positive correlations, orange values are negative correlations.

© 2021 Ganguli I et al. *JAMA Internal Medicine*.

5f. Correlations between measures within drug category

	Antipsychotics for patients with dementia	Opiates for acute disabling low back pain	Antibiotics for acute upper respiratory and ear infections	Two or more concurrent antipsychotic medications	Antibiotics for adenoviral conjunctivitis	Antidepressant monotherapy for bipolar disorder
Antipsychotics for patients with dementia	1					
Opiates for acute disabling low back pain	0.0651	1				
Antibiotics for acute upper respiratory and ear infections	-0.0328	0.3416*	1			
Two or more concurrent antipsychotic medications	0.1947*	-0.0589	-0.2293*	1		
Antibiotics for adenoviral conjunctivitis	-0.035	-0.0507	-0.0569	-0.0306	1	
Antidepressant monotherapy for bipolar disorder	0.0814	0.0906*	0.0689	-0.1046*	0.0744	1

*Represents P-values <0.05. Green denotes measures used in the main composite. Blue values are positive correlations, orange values are negative correlations.

© 2021 Ganguli I et al. *JAMA Internal Medicine*.

eResults. Sensitivity Analyses

Of the systems with greatest relative use of low-value services, all remained among the top thirty users when ranked using Sensitivity Composite 1 that included all 41 measures (Spearman Rank correlation with main composite r, 0.68) (eTable 5). Results using Sensitivity Composite 2 which excluded 5 measures requiring specialized facilities (cardiac catheterization, neurologic testing, vascular testing, and spine surgery) (r, 0.99), and Sensitivity Composite 3 which excluded 3 measures with insufficient history (r, 0.92) were nearly identical.

© 2021 Ganguli I et al. *JAMA Internal Medicine*.

eTable 6. Number of Eligible Beneficiaries and Proportion of Eligible Beneficiaries Whose Attributed Health System Matches the System Associated With NPI on the Low-Value Service Claim (by Measure)[a]

Key Number	Low-value service	Median number of eligible beneficiaries per system (IQR)	NPI system match %
	**Overall (where system of attribution matches system associated with NPI for all low value measure)**		**78.41%**
1	Preoperative laboratory testing	1954 (915, 4484.5)	81.3%
2	Prostate-specific antigen (PSA) testing	1661.5 (797, 3798.5)	83.7%
3	25-hydroxy vitamin D testing	2659.5 (1305.5, 6110)	84.4%
4	Testing for chronic urticaria	108.5 (50, 255.5)	73.7%
5	Immunoglobulin G or E testing	3190 (1545.5, 7582)	63.1%
6	Bleeding time testing	9930.5 (4802, 22805.5)	57.7%
7	Imaging for eye disease	9930.5 (4802, 22805.5)	34.6%
8	Short-interval repeat dual-energy x-ray absorptiometry (DEXA) scan	559 (235, 1241.5)	83.8%
9	Imaging for headache	930.5 (455, 2086.5)	73.8%
10	Carotid artery imaging for simple syncope	1657 (824, 3882.5)	76.1%
11	Head imaging for syncope	588 (299, 1443.5)	64.2%
12	Emergency department head computed tomography (CT) scan for dizziness	1751.5 (872.5, 4207.5)	64.1%
13	Imaging for low back pain	880.5 (435, 1967)	71.1%
14	Head computed tomography (CT) scan for sudden hearing loss	855.5 (374, 2112)	61.8%
15	Imaging for uncomplicated acute rhinosinusitis	2115.5 (996, 5078)	71.7%
16	Magnetic resonance imaging (MRI) for rheumatoid arthritis	40.5 (17, 107)	63.4%
17	Coronary artery calcium scoring for known coronary artery disease (CAD)	2383.5 (1245.5, 5639)	68.6%
18	Dual-energy X-ray absorptiometry (DEXA) scan in low-risk patients	559 (235, 1241.5)	85.5%
19	Screening electrocardiograms (ECGs)	9930.5 (4802, 22805.5)	86.1%
20	Preoperative electrocardiograms (ECG), chest radiographs, or pulmonary function testing (PFT)	8140 (3877, 19406.5)	73.6%
21	Electroencephalography (EEG) for headaches	937 (459.5, 2098)	68.7%
22	Cardiac stress testing	9930.5 (4802, 22805.5)	76.2%
23	Pulmonary function testing (PFT) prior to cardiac surgery	645 (327, 1438)	65.7%
24	Preoperative echocardiography or cardiac stress testing	8255.5 (3991.5, 19893.5)	81.4%
25	Cervical cancer screening	5651.5 (2767.5, 13207)	65.7%
26	Injection for low back pain	1698.5 (858, 4044)	66.7%
27	Repeat short-interval colorectal cancer screening	9930.5 (4802, 22805.5)	82.9%
28	Peripheral access placement without nephrology consultation in stage III-V chronic kidney disease (CKD)	1192.5 (565, 2791.5)	64.6%
29	Feeding tubes for patients with dementia	143 (54, 371)	56.4%
30	Percutaneous coronary intervention (PCI) for asymptomatic patients	533 (270.5, 1129)	72.9%
31	Vertebroplasty for osteoporotic fractures	9930.5 (4802, 22805.5)	68.0%
32	Coronary angiography in low-risk patients	9930.5 (4802, 22805.5)	72.8%
33	Multiple palliative radiotherapy treatments for bone metastases	25.5 (11, 59)	67.6%
34	Renal artery revascularization	6960 (3366, 16402)	63.6%
35	Arthroscopic lavage and debridement for knee osteoarthritis	1375 (670, 3367)	56.8%
36	Antipsychotics for patients with dementia	176.5 (77, 369)	78.5%
37	Opiates for acute disabling low back pain	2190.5 (1103.5, 4952)	75.0%
38	Antibiotics for acute upper respiratory tract and ear infections	2820 (1424.5, 6530)	77.5%
39	Antibiotics for adenoviral conjunctivitis	Suppressed (Suppressed, 13)	65.0%
40	Antidepressant monotherapy for bipolar I disorder	29 (14, 61)	54.9%
41	Two or more concurrent antipsychotic medications	1594.5 (801, 3736)	68.6%

[a]Based on random 20% sample

© 2021 Ganguli I et al. *JAMA Internal Medicine*.
